# Single-cell RNA sequencing uncovers intestinal immune alterations and cellular diversity from chronic fluoride exposure in mice

**DOI:** 10.7150/thno.116567

**Published:** 2025-06-18

**Authors:** Jinge Xin, Yu Chen, Yongmei Huang, Ning Sun, Weiqi Peng, Chunxiao Lai, Ruanruan Yang, Wenqing Chen, Lixiao Duan, Dandan Wang, Yuhao He, Yang Bai, Xueqin Ni, Hesong Wang

**Affiliations:** 1 Department of Gastroenterology, Baiyun District People's Hospital of Guangzhou, Guangzhou, Guangdong, China.; 2 Animal Microecology Institute, College of Veterinary Medicine, Sichuan Agricultural University, Chengdu, Sichuan, China.; 3 Department of Pharmacy, Baiyun District People's Hospital of Guangzhou, Guangzhou, Guangdong, China.; 4 Department of Rehabilitation Medicine, Baiyun District People's Hospital of Guangzhou, Guangzhou, Guangdong, China.

**Keywords:** MHC-Ⅱ signaling pathway, CCL signaling pathway, enterocyte, T cell, plasma cell expansion

## Abstract

**Rationale:**

Chronic exposure to high-fluoride drinking water impairs intestinal structure and function, potentially damaging extraluminal tissues via the gut-organ axis. However, how lifelong exposure to naturally occurring moderate-to-high-fluoride water affects intestinal cells and their underlying mechanisms remain unclear.

**Methods:**

Single-cell RNA sequencing identified cellular heterogeneity and candidate risk genes in the mouse ileum after 56 weeks of 50-ppm fluoride exposure. Cellchart was employed to analyze fluoride-altered cell communication patterns, and gut bacterial richness was ablated using broad-spectrum antibiotics to validate high fluoride-disrupted intercellular signaling pathways.

**Results:**

Fluoride exposure disrupted enterocyte trans-differentiation, affected metabolic health by restricting nutrient absorption, and activated antibacterial activity in enterocytes at the villus base. Downregulation of genes associated with rapid goblet-cell emptying and transmembrane mucin 3 in goblet cell impairs mucus and glycocalyx formation. Antimicrobial peptides, lectins, and lysozymes were reduced in fluoride-exposed Paneth and goblet cells. Fluorescence *in situ* hybridization demonstrated bacterial invasion of the epithelium following mucus barrier damage. Immunologically, fluoride-exposed T cells exhibit high scores for apoptosis, cell cycle suppression, inflammation, and high gut-homing gene expression. Fluoride exposure promoted somatic hypermutation and affinity selection in B-lineage cells while expanding plasma cells with high developmental potential. Ligand-receptor analysis revealed that activated enterocytes presented antigens to T cells via the MHC-II L-R signaling pathway, triggering downstream responses such as upregulating proinflammatory factors and cytotoxic molecules, and remodeling B-lineage cells. Broad-spectrum antibiotics depleted gut microbiota, reducing fluoride-induced gut microbial overgrowth and suppressing MHCII signaling in enterocytes and T/B cell activation—thereby decreasing proinflammatory cytokines and immunoglobulins.

**Conclusions:**

High-fluoride exposure disrupts the intestinal mucus barrier and gut microbiota homeostasis, leading to bacterial invasion of the epithelium that activates MHC-II signaling in absorptive enterocytes. Upregulated MHC-II signaling triggers intestinal immune cell activation and inflammation. These results reveal new intercellular interactions and communication hubs in intestinal cells under fluoride exposure.

## Introduction

Fluoride, the most reactive halogen, is a persistent contaminant. People are exposed to fluoride pollution from various sources: 60% from water, 35% from food, and 5% from the air 1. Approximately 200 million people in 28 countries across the globe are at risk of chronic exposure to elevated concentrations of geogenic fluoride in groundwater 2. Most of those affected reside in Africa (37-46%) and Asia (51-59%) of the total population 3. In China and India, fluoride concentrations in groundwater can reach up to 48 mg L^-1^ 3. Fluoride contamination is widespread across 31 provinces, cities, and autonomous regions in northern and southern China, affecting 59.579 million people from 80,011 villages and 1,049 counties 4. In India, an estimated 62 million people, including 6 million children, suffer from serious health issues due to the ingestion of fluoride-rich groundwater (1.5-39 ppm) 5. Anthropogenic activities such as fertilizer, brick kiln production, glass manufacturing, and aluminum smelting exacerbate fluoride accumulation and pollution 6. Fertilizer production can generate nearly 5.4-11.8 million metric tons of fluoride waste annually 6. The determination of 25 soil samples from aluminum smelting sites in southwestern China showed that the content was as high as 4000 mg F^-^ kg^-1^ 7. Fluoride eventually finds its way into the body through the food chain as plants absorb it from contaminated soil and water 1. According to the World Health Organization guidelines, fluoride exposure levels are categorized as low (0.5-1.0 mg F^-^ L^-1^) and high (>2 mg F^-^ L^-1^) 8.

High-fluoride consumption has adverse effects on health. In addition to dental and bone fluorosis, deleterious effects of fluoride have also been observed in non-bone tissues and organs, such as the brain, kidneys, and gastrointestinal tract 9. Excessive fluoride intake can also induce neurotoxicity, such as spatial memory deficits and dementia 10, 11, by impairing mitochondrial function and biogenesis 12 or suppressing GSK-3b/b-catenin signaling 13. Tian *et al*. 14 found that excessive fluoride exposure upregulated autophagy-related pathways, inducing nephrotoxicity that manifested as disrupted histopathology and ultrastructure. Zhao *et al.* 15 demonstrated that fluoride induces hepatic injury through the IL17a signaling pathway by activating mitochondrial injury and mitophagy. The gastrointestinal tract, particularly the intestine, the primary organ for fluoride absorption, is the first to be affected by fluoride. Yu and Yang 16 evaluated the bio-accessibility of fluoride (F_bio_) in fertilizers using an *in vitro* digestion plasma method. They observed higher F_bio_ in the small and large intestine phases, ranging from 4.35% to 56.33% and 1.01% to 40.52%, respectively. In a study on F_bio_ in polluted soil, Yin *et al.* 7 reported that F_bio_ in the gastric, small intestinal, and colon phases ranged from 2.4% to 48.8%, 2.5% to 38.8%, and 3.9% to 45.7%, respectively. Fluoride exposure can disrupt intestinal morphology, evidenced by decreased and sparse microvilli, reduced villi length, and impaired intestinal barriers 17, 18. Li *et al.* 19 demonstrated that fluoride exposure elevates intracellular calcium ions ([Ca^2+^]_i_), activates the Ca^2+^-dependent RHOA/ROCK pathway, and triggers MLC2 phosphorylation and rearrangement of ZO-1 and F-actin, which are the underlying mechanisms of intestinal barrier disruption. Sun *et al.* 20 reported that excessive fluoride exposure reduced digestive enzyme activity (amylase, trypsin, and lipase) and goblet cell number while increasing secretory IgA (sIgA) levels in the mouse jejunum and ileum. Gao *et al.* 21 similarly reported a decrease in goblet cells and noted that fluoride exposure inhibited the proliferation capacity of intestinal epithelia, disturbed the T helper (Th) 17 cell-related cytokine expression, decreased the intestinal IL17a levels, and increased the intestinal IL-1β, IL-23, and IL-22 levels. Zhang *et al.* 22 demonstrated that the TLR/NF-κB pathway activation is the key mechanism of intestinal inflammation in fluorosis mice. Wu *et al.* 23 suggested that bile acid metabolism is involved in fluoride-induced ileal toxicity; reducing the levels of cholic acid transporters, ASBT, IBABP, OST-α, and OST-β, in the ileum can alleviate this toxicity. Dionizio *et al.* 24 found that fluoride exposure caused gastrointestinal symptoms (such as vomiting with blood and diarrhea) accompanied by a decrease in nNOS-IR and HuC/D-IR neuronal density and increased tunica muscularis thickness and myosin isoforms in the small intestine. Additionally, proteomic analysis showed reduced energy metabolism-related proteins in the rat ileum after acute fluoride exposure 24. Li *et al.* 25 found that mice exposed to fluoride for 90 days showed damaged intestinal permeability, upregulated inflammatory response, and increased pyroptosis-related protein levels, which recovered after 30 days. Thus, intestinal cells facilitate digestion and nutrient absorption, provide a barrier against harmful substances and organisms, and possess significant immune properties.

Although studies on fluoride intestinal toxicity have been conducted to some extent, the impact and mechanism of lifelong exposure to naturally occurring moderate-to-high-fluoride drinking water on intestinal function remain poorly characterized. It is, therefore, necessary to comprehensively understand how prolonged fluoride exposure damages intestinal cells, particularly the specific contributions of each cell subtype. Single-cell RNA sequencing (scRNA-seq) can identify the target cell types of pollutants and distinguish the underlying transcriptome heterogeneity among cell types. scRNA-seq has also provided a deeper understanding of the cellular responses in digestive diseases like inflammatory bowel disease, celiac disease, and colon cancer 25, 26. For instance, Huang *et al.* 26 used scRNA-seq to show the involvement of the cyclic adenosine monophosphate response pathway in the pathogenesis of pediatric-onset colitis, identify multiple cellular defects, and demonstrate that the phosphodiesterase (PDE) inhibitor, dipyridamole, successfully ameliorated clinical symptoms in both mice and children with colitis. Similarly, Kong *et al.* 27 used scRNA-seq to study active Crohn's disease, revealing dominant transcriptional alterations in stromal and epithelial cells and identifying markers of disease-associated myofibroblast activation. scRNA-seq offers a more profound understanding of the mechanisms of intestinal damage based on fluoride exposure-associated changes. Understanding the contributions of different cell populations to intestinal epithelial impairment following fluoride exposure is important for developing therapies that help heal and restore normal intestinal function.

In this study, male mice were exposed to 50 ppm fluoride in drinking water for 56 weeks, and scRNA-seq was used to define transcriptional states, cellular heterogeneity, immune characteristics, and cell crosstalk at single-cell resolution in the ileum. The aim was to provide a transcriptional landscape of fluoride-exposed intestines at single-cell resolution, identify candidate risk genes potentially contributing to disease development, and thereby facilitate the uncovering of disease pathogenesis and therapeutic targets.

## Experimental section

### Animal maintenance

Experiment 1: Male C57BL/6J mice (3 weeks old) were provided by Si Pei Fu (SPF Biotechnology Co. Ltd., Beijing, China) and housed in cages. After one week of acclimatization, the mice were provided normal (Ctrl group, n = 12) and high-fluoride drinking water (50 ppm) for 56 weeks (F group, n = 12). During the experimental period, the animal lab maintained a controlled temperature (20-22 °C) and a 12-h light/dark cycle. Mice had *ad libitum* access to standard food.

Experiment 2: Intestinal flora clearance: Male C57BL/6J mice (3 weeks old) were provided by Si Pei Fu and housed in cages. After one week of acclimatization, the mice were randomly divided into four groups: control group (Ctrl group, n = 10), fluoride exposure group (F group, n = 10), antibiotic administration group (Anti group, n = 10), and antibiotic administration coupled with fluoride exposure group (Anti+F group, n = 10). The Ctrl and F groups were provided with normal drinking water, and gavaged with ultrapure water or high fluoride solution (dissolved in ultrapure water, 24 mg kg^-1^, 0.2 mL), respectively, for eight weeks. Mice in the Anti and Anti+F groups were intermittently provided with antibiotic-containing water (two weeks of antibiotic-containing water followed by two weeks of normal water), and gavaged with ultrapure water or high fluoride solution (dissolved in ultrapure water, 24 mg kg^-1^, 0.2 mL), respectively, for eight weeks. Antibiotic-containing water: ciprofloxacin (0.2 g L^-1^; Rhawn, China) and metronidazole (1 g L^-1^, Rhawn, China). During the experimental period, the animals were maintained at a controlled temperature of 20-22 °C and a 12-h light/dark cycle. Mice had *ad libitum* access to standard food.

At the end of the experiment, three mice from each group in Experiment 1 were euthanized after fasting for 12 h and used for single-cell isolation and sequencing. The remaining mice from Experiments 1 and 2 were sacrificed, and their ileal tissues with and without contents were collected. A segment of the ileum containing luminal contents from Experiment 1 was dissected, stored at -80 °C for fluorescence *in situ* hybridization in frozen sections. A segment of the ileum without luminal contents form Experiment 1 and 2 were dissected and fixed in 4% paraformaldehyde for Hematoxylin-eosin staining, AB-PAS staining, immunofluorescence and immunohistochemistry. A segment of the ileum form Experiment 1 and 2 were dissected rinsed with cold, sterile saline, blotted with absorbent paper, quickly frozen in liquid nitrogen, and stored at -80 °C for Enzyme-linked immunosorbent assay. All experiments were conducted in accordance with the Guidelines for the Care and Use of Laboratory Animals and were approved by the Institutional Animal Care and Use Committee of Sichuan Agricultural University (approval number: SYXKchuan2019-187).

### Generation and sequencing of single-cell libraries

#### Single-cell isolation

The terminal ileum was excised, gently dissected longitudinally, and rinsed with cold Dulbecco's phosphate-buffered saline (DPBS; n = 3 per group). The tissues were gently cut into small pieces, transferred to a 50-mL centrifuge tube, and incubated with 5 mL collagenase (type Ⅳ) digestion solution at 37 °C in a water bath for 45 min to obtain dissociated single cells. The single-cell suspension was filtered through a 40-µm strainer. The remaining cells were pelleted by centrifugation (1,000 rpm for 5 min at 4 °C), resuspended in cold DPBS (without Ca^2+^ and Mg^2+^, but containing 2% fetal bovine serum [FBS]), and re-centrifuged (1,000 rpm for 5 min at 4 °C). The cell pellet was collected, and 3 mL of red-blood-cell lysis buffer was added and incubated for 5 min to remove the erythrocytes. After another centrifugation (1,000 rpm for 5 min at 4 °C), the cell pellet was resuspended in cold DPBS (containing 2% FBS). Cell counts and viability, which were ≥ 80%, were analyzed using a fluorescence cell analyzer or hemocytometer, and cell concentration was adjusted.

#### scRNA-seq

The single-cell suspensions were converted to barcoded scRNA-seq libraries using the Chromium Next GEM Single Cell 3' Reagent Kits v3.1 (Dual Index; 10 × Genomics, USA) following the manufacturer's instructions. Briefly, the cell suspensions were loaded onto a 10 × Chromium Single Cell Platform (10 × Genomics) at a concentration of 1,000 cells per μL to generate Gel Beads-in-emulsion (GEMs) combining barcoded Single Cell 3' v3.1. The barcoded GEMs were removed from the Recovery Wells and subjected to reverse transcription (RT) in a thermal cycler using the following protocol: 53 °C for 45 min, 85 °C for 5 min, and 4 °C indefinitely. First-strand cDNA was purified from the post-GEM-RT reaction mixture using silane magnetic beads. Barcoded, full-length cDNA was then PCR-amplified to generate sufficient mass for 3' gene expression library construction. The final library pool was sequenced on an Illumina Nova6000 instrument (Shanghai Personalbio, Shanghai, China) using 150 base-pair paired-end reads.

### Bioinformatic analysis of scRNA-seq data

#### Processing single-cell transcriptomics data and quality control

Mapping reads for the GRCm38 mouse genome, quality control, and counting reads for Ensembl genes were performed using Cell Ranger V6.0 software (10 × Genomics). Downstream analysis of these datasets was performed using Seurat v.4.9.0. For quality control, the following filters were applied: (i) genes expressed in ≤ 3 cells were excluded; (ii) cells expressing >5000 genes (potential doublets) or < 200 genes (low quality) were excluded from further analysis; (iii) cells with Unique Molecular Identifier of < 25000 were excluded from further analysis; (iv) cells with > 15% of genes derived from the mitochondrial genome were not considered; (v) doublets were identified and removed using the DoubletFinder package.

#### Clustering and identification of cell types

Clustering was performed using the top 2,000 variable genes selected using the FindVariableFeatures function for principal component analysis (PCA). Cells were clustered using the top 20 principal components selected by PCA based on highly variable genes, using the FindClusters function with the Louvain algorithm and a resolution parameter of 0.8. Batch effects between samples were corrected using Harmony. The resulting cell clusters were visualized using the Uniform Manifold Approximation and Projection (UMAP) method and annotated by examining the expression of known marker genes.

#### Differential expression and functional enrichment analysis

Differentially expressed genes (DEGs) were identified using Seurat's FindMarkers function, based on the Wilcox likelihood-ratio test with default parameters. DEGs were selected if expressed in > 25% of the cells in a cluster with an average log (fold change) value of > 0.25. To investigate the potential functions of DEGs, the Gene Ontology (GO), Reactome, and Kyoto Encyclopedia of Genes and Genomes (KEGG) analyses were used via the “clusterProfiler” R package, considering pathways with p_adj values of < 0.05 as significantly enriched.

#### Trajectory analysis

Pseudo-time trajectory analysis to map the differentiation or conversion of particular cell types was performed using Monocle2 28. The FindVariableFeatures function of Seurat was used to select highly variable genes from clusters. Subsequently, DDRTree was used to perform dimension reduction, and cell trajectories were visualized through plotting.

#### Definition of cell scores and signature

The UCell R package was used to evaluate gene signature enrichment based on the scRNA-seq data 29. The gene expression matrix was used to calculate signature scores for each cell in each gene set. UCell scores were based on the relative ranking of genes in individual cells.

#### Cell communication

Cell-cell interactions were evaluated using CellChat (v2, R package) 30. CellChat uses gene expression data as user input to model cell-cell-communication probabilities by integrating gene expression with an existing database consisting of known interactions among signaling ligands, receptors, and cofactors. In this study, cell-cell interactions were analyzed individually under different conditions following the default pipeline. Normalized count data from each condition were used to create the CellChat object, and the recommended preprocessing functions were applied to analyze individual datasets with default parameters.

### Hematoxylin-eosin staining

Ileum samples, devoid of contents, were fixed in 4% paraformaldehyde and prepared as paraffin sections. As described previously 31, the sections were stained with hematoxylin and eosin. Images were captured using an OLYMPUS microscope (OLYMPUS, Japan).

### AB-PAS staining

Paraffin-embedded sections of ileal tissue without contents were prepared, dewaxed in water, rewarmed, and fixed as frozen sections. The sections were stained with AB-PAS dye solution C (AB-PAS C) for 15 min, rinsed with tap water until colorless, stained with AB-PAS B for 15 min, and rinsed once with tap water and twice with distilled water. Subsequently, the sections were stained with AB-PAS A for 30 min at room temperature in the dark, rinsed for 5 min, dehydrated, and sealed. Images were captured using an OLYMPUS microscope.

### Immunofluorescence and immunohistochemistry

After fixation in 4% paraformaldehyde, ileum samples without contents were embedded in paraffin and cut into 3-μm-thick sections. Double-label immunofluorescence, single-label immunofluorescence, and immunohistochemistry were performed as previously described 31. Primary antibodies diluted in PBS included: anti-DUOX (1:200, BS-11432R, Bioss, Beijing, China), anti-CD8 (1:2000, GB15068, Servicebio, Wuhan, China), anti-ITGAE (1:2000, 65047-1-Ig, Proteintech, Wuhan, China), anti-CD4 (1:2000, GB15064, Servicebio), anti-CCR9 (1:3000, BS-1788R, Bioss), anti-CD226 (1:3000, ER63273, HUABIO, Hangzhou, China), anti-CCR2 (1:4000, GB11326, Servicebio), anti-MUC2 (1:3000, GB11344-100, Servicebio), anti-RAMP1 (1:3000, 1037-1-AP, Proteintech), anti-REG3G (1:3000, K003255P, Solarbio, Beijing, China), anti-EPCAM (1:3000, 21050-1-AP, Proteintech), anti-ACE2 (1:5000, GB11267-100, Servicebio), anti-LYZ1 (1:500, GB11345-100, Servicebio), anti-H2-Ab1 (1:4000, GB113146-100, Servicebio), anti-4-HNE (1:100, BS-6313R, Bioss) and Phalloidin-iFluor® 594 Conjugate (1:1000, Catalog number: 23122, AAT Bioquest, Pleasanton, CA 94588, USA).

### Fluorescence *in situ* hybridization in frozen sections

Ileal tissues containing luminal contents were retrieved from a -80 °C freezer, embedded in optimal cutting temperature (OCT) compound, and equilibrated at -40 °C for cryosectioning. Frozen sections were dried at room temperature and fixed with 4% paraformaldehyde (DEPC) for 10 min. Proteinase K (20 μg mL^-1^) was added to digest the tissues. Hybridization buffer was added, and specimens were incubated at 37 °C for 1 h. After removing the pre-hybridization solution, the probe hybridization solution (1 µM) was added, and sections were incubated in a humidified chamber and hybridized overnight at 40 °C. SSC solution (Servicebio) was used to wash the sections and remove non-specific hybrids. After blocking with serum, PBS containing MUC2 primary antibody (1:100) was added and incubated at 4 °C overnight. Sections were then incubated with a Cy5 secondary antibody for 50 min at room temperature. PBS containing Phalloidin-iFluor^®^ 594 Conjugate (1:1000) was added and incubated at 4 °C overnight. Sections were incubated with DAPI for 8 min in the dark and mounted. An EUB338 probe (5′-GCTGCCTCCCGTAGGAGT-3'), labeled 5′ with Alexa 647, was used.

### Enzyme-linked immunosorbent assay

Ileal tissue was ground to a 10% homogenate with 1 × PBS and centrifuged at 3,000× *g* for 10 min to collect the supernatant. IL-17a, IFN-γ, IL-21, α-DEFENSIN, GZMA, and GAMB levels were detected using the corresponding enzyme-linked immunosorbent assay (ELISA) quantification set (mLbio, Shanghai, China) according to the manufacturer's instructions.

Quantification of immunoglobulins was performed, consistent with Tan *et al.* 32. Fecal samples were resuspended in 1 × PBS (100 mg mL^-1^) with protease inhibitor cocktail (AR1182, Boster, Wuhan, China), centrifuged at 14,000 × *g* for 10 min to pellet the bacteria and debris, and the supernatant was collected. sIgA and secretory IgM (sIgM) levels were detected using mouse sIgA or sIgM ELISA quantification kits (mLBio, Shanghai, China), according to the manufacturer's instructions.

### 16S rRNA gene sequencing

The ileal content of the mice from Experiment 2 was collected for 16S rRNA gene sequencing. The detailed method has been described previously 33.

### Quantification and statistical analysis

RNA-seq statistical analysis is described in the Methods section. All other statistical analyses were presented as the mean ± standard deviation and performed using IBM SPSS 25. Unless otherwise specified in the figure captions, *p*-values were determined using Student's two-tailed t-tests for pairwise comparisons between groups.

## Results

### Fluoride exposure alters the percentage and transcriptome of intestinal cells across different cell types

**Figure 1:**
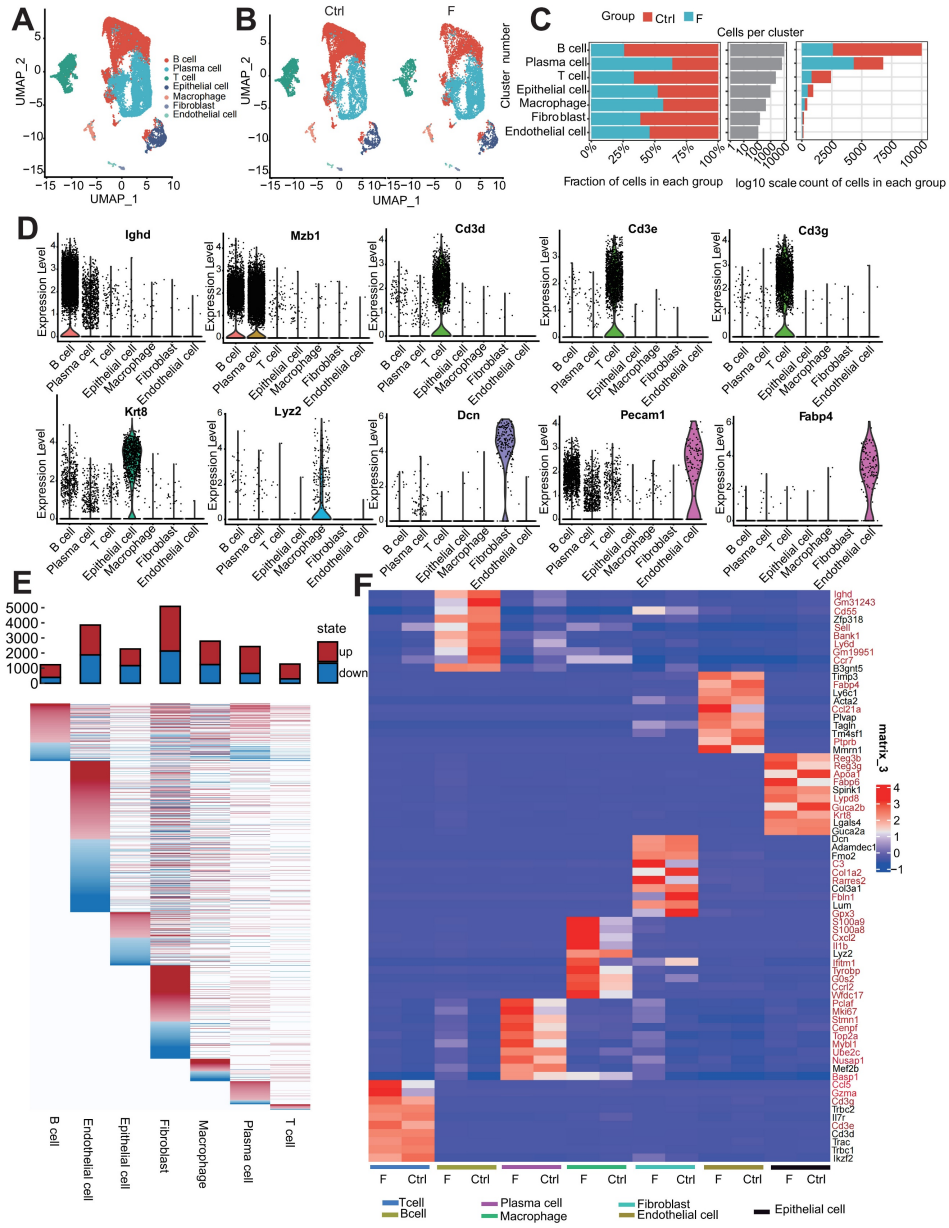
Fluoride exposure alters the percentage of intestinal cells across different cell types. (A) UMAP of 20,904 intestinal cells from unexposed and exposed mice. (B) UMAPs of 12,310 intestinal cells from unexposed (left) vs. 8,594 cells from exposed animals (right). (C) Difference in the percentage of cells and cell count per cluster (unexposed-exposed). (D) Expression of representative selected genes in clusters from (A). (E) DEGs for each intestinal cell type. The top bars indicate the DEG count (red for upregulated and blue for downregulated; to avoid concealing relatively small differentially varying genes in the visualization, the value of |log_2_FC|≥1 was uniformly recorded as 1). (F) Heatmap showing the expression of the top 10 marker genes of each subtype in the Ctrl and F groups. Red indicates significant differences between the groups.

To investigate intestinal cell responses to fluoride exposure, ileal intestinal cells were isolated from control adult mice and mice exposed to fluoride. In the initial survey, 12,310 and 8,594 cells were sorted from the ileum of the Ctrl group (provided with normal water) and F groups (provided with high-fluoride drinking water [50 ppm] for 56 weeks), respectively. Gene expression analysis of individual ileal cells from fluoride-exposed and unexposed mice revealed seven distinct cell clusters with unsupervised clustering using UMAP (Figure 1A). These clusters were annotated based on the differential expression of marker genes into B cells (marker gene, *Ighd*), plasma cells (PCs; marker gene, *Mzb1*), T cells (marker genes, *Cd3d*, *Cd3e,* and *Cd3g*), epithelial cells (marker gene, *Krt8*), macrophages (marker gene, *Lyz2*), fibroblasts (marker gene, *Dcn*), and endothelial cells (marker gene, *Fabp4*) (Figure 1D).

Clustering of each cell type was compared between the control and fluoride-exposed mice to identify the presence of global changes (Figure 1B). All cell subtypes were detected in both groups, though in varying proportions; this result indicates that fluoride exposure did not lead to the loss or acquisition of any specific intestinal cell type (Figure 1B).

Among non-immune cells, fibroblasts were less abundant in fluoride-exposed mice than in the control group, and epithelial and endothelial cells were present in similar proportions in both groups (Figure 1B-C). Among the identified immune cells, the proportions of T and B cells were lower, whereas the PC and macrophage infiltration levels were relatively higher in mice in the F group than those in mice in the Ctrl group (Figure 1B-C), indicating substantial heterogeneity of immune cell composition due to fluoride exposure. The numbers of B and effector B cells (PCs) were the most affected by fluoride exposure (Figure 1B-C).

A heatmap of seven intestinal cell-subset DEGs showed varied DEG features in quantity and pattern (Figure 1E). Fibroblasts had the highest number of DEGs (2,953 upregulated and 2,135 downregulated), followed by endothelial cells (1,977 upregulated and 1,877 downregulated), macrophages (1,552 upregulated and 1,236 downregulated), PCs (1,777 upregulated and 653 downregulated), epithelial cells (1,104 upregulated and 1,168 downregulated), T cells (978 upregulated and 293 downregulated), and B cells (837 upregulated and 389 downregulated). Aberrant expression of cell marker genes can indicate their involvement in disease development. Inter-group comparisons of cell top 10 marker gene expression demonstrated that high fluoride exposure significantly altered the expression of the marker genes in various cell types. (Figure 1F). Single-cell analysis revealed aberrant proportions and transcriptome heterogeneity across cell types, suggesting altered intestinal homeostasis following fluoride exposure.

### Fluoride exposure influences normal enterocyte differentiation

**Figure 2:**
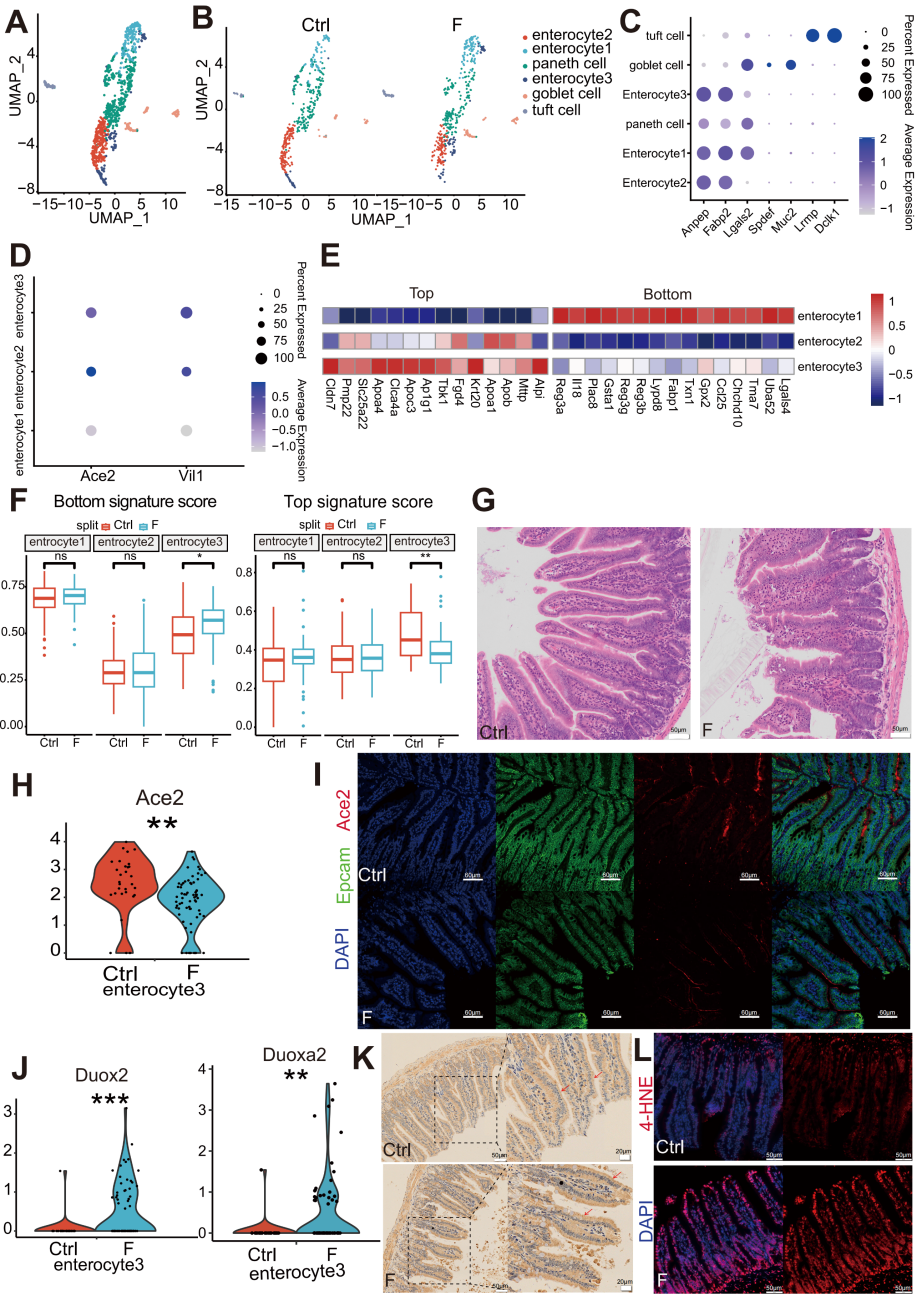
Fluoride exposure inhibits enterocyte differentiation in the villi middle. (A) UMAP of sub-clustered epithelial cells from unexposed and exposed mice. (B) UMAPs of unexposed (left) vs. exposed (right) samples. (C) Bubble chart showing the distribution and expression of selected representative genes in clusters from (A). (D) Bubble chart showing the distribution and expression of mature marker genes in the enterocyte subsets. (E) Heatmap indicating the expression of selected gene sets in enterocyte subsets. (F) Box plot displaying the expression scores of the bottom and top genes in enterocyte subtypes from control and fluoride samples. (G) Representative HE-stained images of ileum tissues from control and fluoride-exposed mice (n = 3 biological replicates). (H) Violin plot showing the expression of *Ace2* in enterocyte subtypes from control and fluoride samples. P-values were calculated using the Wilcoxon test. (I) Representative immunofluorescence (IF) staining images of ACE2 in ileum tissues from control and fluoride-exposed mice (n = 3 biological replicates). Epcam labels the cytoskeleton of intestinal epithelial cells. Red represents the ACE2-positive cells. (J) Violin plot showing *Duox2* expression in enterocyte subtypes from control and fluoride-treated samples based on scRNA-seq. P-values were calculated using the Wilcoxon test. (K) Representative immunohistochemical staining images of DUOX2 in ileal tissues from control and fluoride-exposed mice (n = 3 biological replicates). The red arrows indicate the presence of DUOX2 protein. (L) Representative immunohistochemical staining images of 4-HNE in ileal tissues from control and fluoride-exposed mice (n = 3 biological replicates). *, *p* < 0.05; **, *p* < 0.01; ***, *p* < 0.001; ****, *p* < 0.0001 were considered significant, and ns signifies non-significant differences.

UMAP analyses were performed to further characterize cell populations within epithelial cell clusters. Epithelial cells comprise six different cell lineages that can be classified into two broad categories: absorptive and secretory. Absorptive cells include enterocytes with nutrient- and water-absorbing functions and microfold cells (M cells), which sample the gut contents and transport luminal antigens to potential immune cells that comprise Peyer's patches (PP). Secretory cells include Paneth, goblet, enteroendocrine, and tuft cells. Six clusters were identified within the epithelial cell population: three clusters of absorptive cells (enterocyte clusters 1, 2, and 3) and three clusters of secretory cells (Paneth, goblet, and tuft cells) (Figure 2A). The Ctrl and F groups shared all cell subtypes, though in different proportions (Figure 2B and Figure S1A). The classification was based on the differential expression of cell-type marker genes (Figure 2C**)**.

Markers of mature enterocytes, ACE2 34 and Vil1 35, which are brush border proteins, were highly expressed in enterocyte clusters 2 and 3 (Figure 2D). Conversely, enterocyte cluster 1 exhibited low expression of these markers (Figure 2D). In line with this, Monocle2 analysis indicated that enterocyte cluster 1 was the least differentiated, enterocyte cluster 2 was highly differentiated, and enterocyte cluster 3 represented a middle transition state (Figure S1B). These expression patterns of genes associated with mature enterocyte function combined with the results of Monocle2 analysis highlighted variations in the differentiation and maturation among the enterocyte clusters. Enterocytes are not terminally differentiated cells upon emergence from crypts but continue to transdifferentiate along the villus axis, which allows them to acquire different functions at various positions 36.

The expression of zonated genes in the three enterocyte clusters was further assessed to distinguish their location within the intestinal villi based on previously reported zonated genes. 34-37. The enrichment of bottom villi genes (*Reg3a*, *Il18*, and *Plac8*) was observed in enterocyte cluster 1, top villi genes (*Apoa1*, *Apob*, and *Mttp*) in enterocyte cluster 2, and a mixed signature of both spatial markers in enterocyte cluster 3 (Figure 2E). These expression patterns indicate that enterocyte cluster 1 is located at the villus base, enterocyte cluster 2 at the villus top, and enterocyte cluster 3 at the villus middle. Moreover, the differentiation pattern of the three clusters of enterocytes represented by the zonated genes was consistent with the results of the Monocle2 analysis.

To assess the differentiation features of enterocytes exposed to fluoride, differences in the module scores of the zonated landmark genes in enterocytes were evaluated between the Ctrl and F groups. Compared with that in the Ctrl group, enterocyte cluster 3 with fluoride exposure exhibited higher villus-bottom and lower villus-top feature scores (Figure 2F), suggesting an inhibition of normal differentiation. Inhibition of trans-differentiation and maturation of enterocyte cluster 3 with fluoride exposure may result in long-term deficiency of mature cell replenishment after the removal of enterocyte cluster 2 at the top, resulting in a defect at the top of the villi. Hematoxylin-eosin (HE) staining verified the presence of a defect at the top of the villi in fluoride-exposed ilea compared with those of the Ctrl group (Figure 2G). The villus expression pattern of the mature enterocyte marker *ACE2* in enterocyte cluster 3 was further examined. Consistent with the villus-bottom feature score, *ACE2* expression was significantly downregulated in enterocyte cluster 3 following fluoride exposure (Figure 2H). Immunofluorescence confirmed lower ACE2 levels in enterocytes located in the middle of the villi in the F group than those in the Ctrl group (Figure 2I).

Enterocytes approaching the top of the villi present greater differentiation 35. Reduced mature enterocyte signatures are common in various inflammatory and infectious small bowel pathologies. Among the top 15 most highly upregulated genes, enrichment of the epithelial antimicrobial dual oxidase *Duox2* and its maturation partner *Duoxa2* was observed in enterocyte cluster 3 of fluoride-exposed samples (Figure 2J). MacFie *et al.* 38 verified that *Duox2* and *Duoxa2* maturation factors form the prevalent enzyme system capable of producing the reactive oxygen species H_2_O_2_ in active ulcerative colitis. Immunohistochemical staining verified high DUOX2 levels in enterocytes of the villi and increased lipid peroxidation in the F group in immunofluorescence experiments (Figure 2K-L).

### Fluoride exposure disrupts nutrient transporters and antimicrobial function in enterocytes, spatially zonated along the villus axis

**Figure 3:**
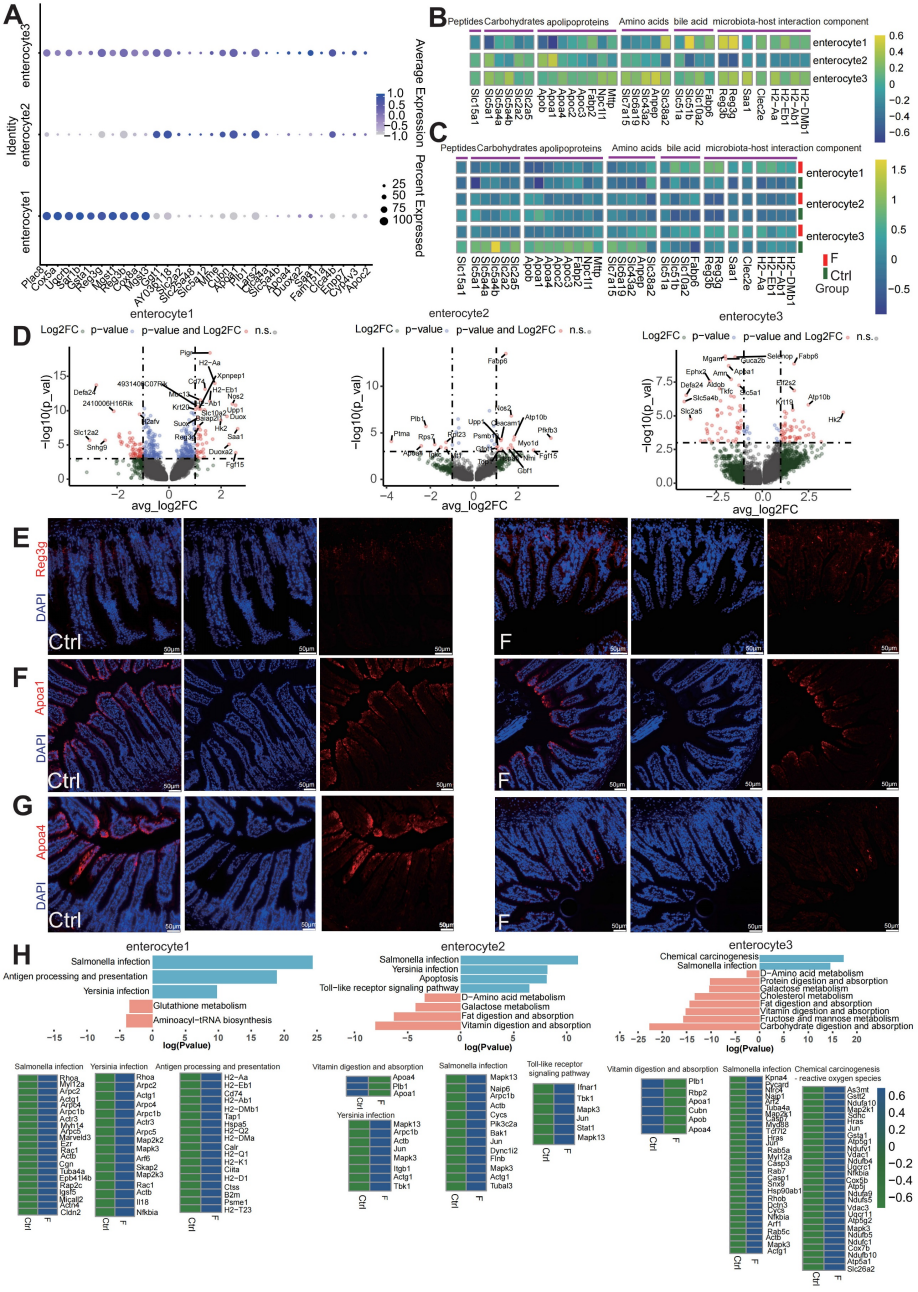
Fluoride exposure disrupts the absorption of enterocytes while activating their antimicrobial activity. (A) Bubble chart showing the expression features of enterocyte zonation markers at the bottom and top of the villus in enterocyte subtypes. (B) Heatmap indicating the expression of selected gene sets in enterocyte subsets. (C) Heatmap showing the differential expression of selected gene sets in control and fluoride-exposed enterocyte subsets, including the transport of peptides, carbohydrates, apolipoproteins, amino acids, bile acids, and microbiota-host interaction components. (D) Volcano plot showing differentially expressed genes between control and fluoride-exposed enterocyte subtypes. The names of the most significant genes are shown in the plots. Representative immunofluorescence staining images for APOA1 (E), APOA4 (F), and REG3G (G) in ileum tissues from control and fluoride-exposed mice (n = 3 biological replicates). (H) Two-sided bar graph showing the inhibition and activation pathways enriched in enterocytes in the F group according to KEGG based on downregulated (left) or upregulated (right) genes upon switching to fluoride exposure. The selected KEGG pathways were used to identify the genes involved.

Enterocytes, the dominant villus epithelial cell type, are responsible for nutrient absorption and protecting the body from harsh bacteria-rich environments. Analysis of the top DEGs among the three enterocyte clusters revealed that villus-top enterocytes (enterocyte cluster 2) exhibited higher expression of genes involved in facilitating nutrient transportation, villus-bottom enterocytes (enterocyte cluster 1) displayed higher expression of genes associated with immunoregulatory and antimicrobial functions, whereas villus-middle enterocytes (enterocyte cluster 3) combined these features (Figure 3A). These results suggest that the expression pattern of functional genes along the villus axis reflects a transition from antibacterial gene functions at the bottom to a nutrient-absorption function at the top of the villus, consistent with previous research 36.

The ubiquitous spatial transcriptional heterogeneity of differentiated enterocytes resulted in the zonation of nutrient transporters, most of which are located at the top and middle villi, except for bile acid uptake, which was located at the villus bottom (Figure 3B). The expression of key nutrient transporters was examined to better understand the nutrient-absorption features of enterocytes in the fluoride-exposed intestine. Significantly downregulated was observed in the ecarbohydrate transporters (*Slc5a1*, *Slc5a4a*, *Slc5a4b*, *Slc2a2*, and *Slc2a5*), apolipoproteins associated with lipoprotein biosynthesis and vitamin digestion and absorption (*Apoa1*, *Apoa4*, *Apob*, *Apoc3*, *Fabp2*, *Npc1l1*, and *Mttp*), the main peptide transporter *Slc15a1*, and amino acid transporters (*Slc6a19*, *Slc7a15*, *Slc43a2*, and *Anpep*) in fluoride-exposed top and middle enterocytes, particularly in enterocyte cluster 3.

Conversely, bile acid transporters (*Slc51a*, *Slc51b*, *Slc10a2*, and *Fabp6*) were enriched across all fluoride-exposed enterocyte clusters (Figure 3C and Table S1). DEG analysis also showed that the majority of the genes with the most significant changes belonged to nutrient transporters in enterocyte clusters 2 and 3 (Figure 3D). Immunofluorescence verified that APOA1 (Figure 3E) and APOA4 (Figure 3F) levels were significantly reduced in enterocytes located in the middle and top of the villus of the F group compared with their levels in the Ctrl group.

KEGG pathway enrichment analysis showed downregulation of pathways for vitamins, carbohydrates, protein, and fat digestion and absorption, D-amino acid, galactose, and cholesterol metabolism, as well as fructose and mannose metabolism in villus-top enterocytes (enterocyte clusters 2 and 3) (Figure 3H). Conversely, the bile secretion pathway was upregulated in the villus-bottom enterocytes (enterocyte cluster 1).

Dysfunction of *Slc* family genes, which are major nutrient transporters, is linked to numerous diseases 39, 40. The expression patterns of *Reg* family members and other components related to microbiota-host interactions were further tested in the bottom villus cells of fluoride-exposed mice. Fluoride exposure dramatically increased the expression levels of the antimicrobial peptide genes *Reg3b* and *Reg3g*, enterocyte inflammatory protein *Saa1* 41, bacterial pathogen adherence protein *Clec2e* 42, and major immune-related MHC class II genes (*H2-Aa, H2-Eb1, H2-Ab1*, and *H2-DMb*) in villus-bottom enterocytes (enterocyte cluster 1) (Figure 3C). These findings indicate that fluoride exposure activates the antimicrobial program in the villus-bottom enterocytes, which are gatekeepers for crypt stem cells to avoid pathogenic microbial exposure 36. DEG analysis highlighted that the majority of the genes with the most significant changes belonged to those associated with microbiota-host interactions in enterocyte cluster 1 (Figure 3D). Immunofluorescence also demonstrated a significant increase in the antimicrobial peptide REG3G in fluoride-exposed samples compared with controls (Figure 3G**)**. Furthermore, KEGG pathway enrichment analysis of upregulated DEGs indicated strong upregulation of pathways related to pathogen infection, including *Yersinia*, *Salmonella*, antigen processing and presentation, and TOLL-like receptor signaling, in fluoride-exposed villus-bottom enterocytes (enterocyte cluster 1) (Figure 3H).

### Fluoride exposure damages the intestinal mucus and reduces antibacterial substance secretion, enabling bacteria to invade the intestinal epithelium

**Figure 4:**
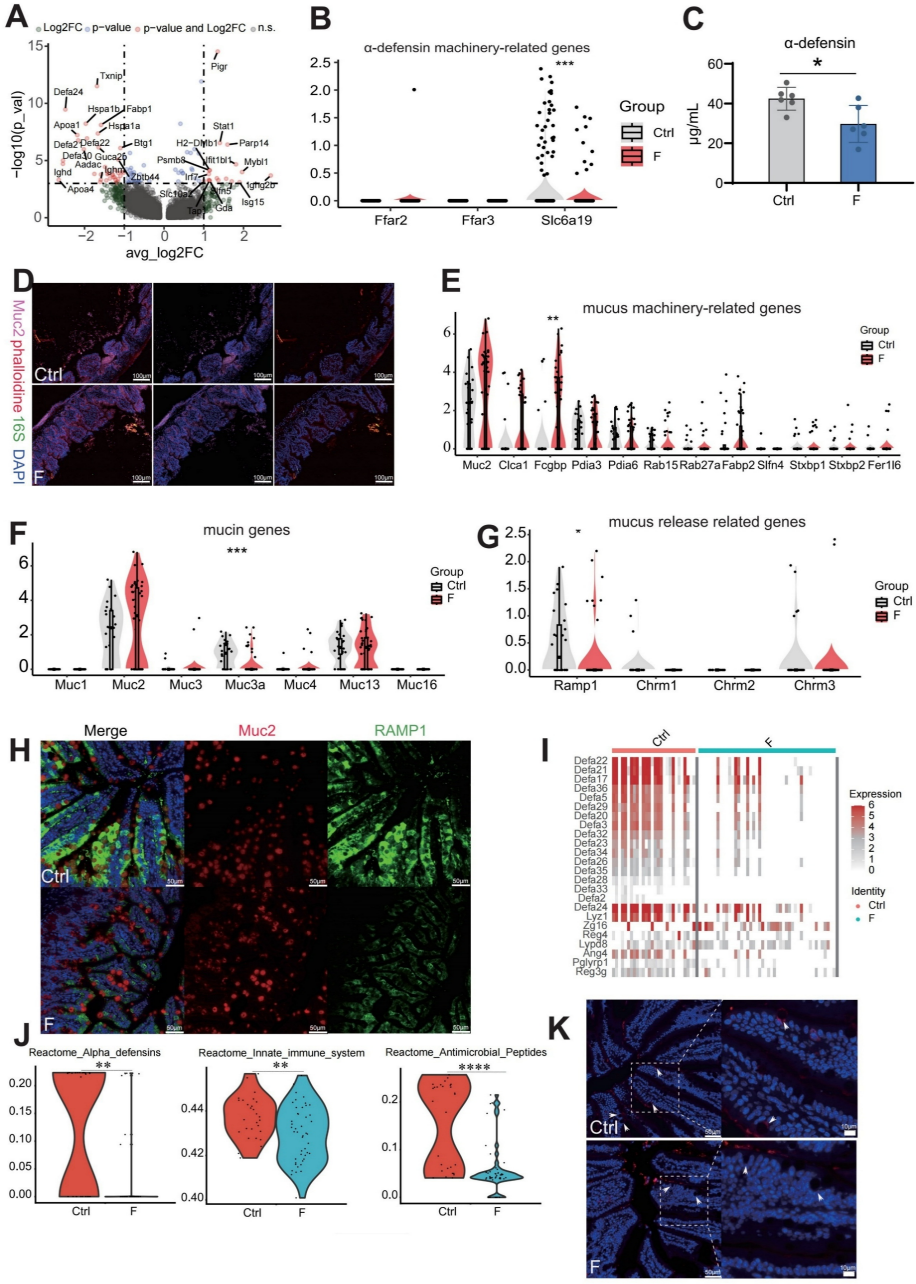
Fluoride exposure inhibits the secretion of antibacterial substances and intestinal mucus emptying. (A) Volcano plot showing differentially expressed genes between normal and fluoride-exposed Paneth cells. The names of the most significant genes are shown in the plots. (B) Violin plot indicating the expression of α-DEFENSIN machinery-related genes in ileal Paneth cells from control and fluoride-exposed mice. Significance was determined using the Wilcoxon test. (C) α-DEFENSIN content in the ileum, detected by ELISA. (D) Analysis of bacteria-mucus-epithelial localization in the ileum. Phalloidine stains the intestinal cytoskeleton, the 16S rRNA fluorescent probe labels bacteria, and MUC2 labels mucus. White arrows mark bacteria invading the intestinal epithelium. Violin plots indicate the expression of mucus machinery-related genes (E), mucin genes (F), and mucus release-related genes (G) in ileal goblet cells from control and fluoride-exposed mice. Significance was determined using the Wilcoxon test. (H) Immunofluorescence staining of RAMP1 and MUC2 in the mouse ileum (n = 3 biological replicates). (I) Heatmap representing the expression of selected genes in goblet cells from the Ctrl and F groups. (J) Violin plots indicate the enriched inhibitory pathways in goblet cells from the F group as determined by reactome analysis. (K) Representative immunofluorescence-staining images of LYZ1 in ileal tissues from control and fluoride-exposed mice (n = 3 biological replicates). White arrows mark the positive expression of LYZ1. *, *p* < 0.05; **, *p* < 0.01; ***, *p* < 0.001; ****, *p* < 0.0001 were considered significant, and ns denotes non-significant differences.

Small-intestinal Paneth cells act as gatekeepers of gut innate immunity by producing and secreting antimicrobial peptides 43. Paneth cell dysfunction or defects can lead to intestinal inflammation, as seen in inflammatory bowel disease models 43. scRNA-seq identified genes coding for α-defensins (*Defa21*, *Defa22*, *Defa24*, and *Defa30*) in Paneth cells as the most downregulated by fluoride exposure (Figure 4A). Reduced α-DEFENSIN protein levels in the ileum were verified by ELISA (Figure 4C). Immunoglobulin (Ig) M generated by Paneth cells was significantly reduced in fluoride-exposed mice than in control mice (Figure 4A).

Nutrient absorption, particularly butyric acid and leucine, is crucial for driving α-DEFENSIN secretion by Paneth cells 44. Therefore, expression levels of the transporter-encoding genes for butyric acid (*Ffar2* and *Ffar3*) and leucine (*Slc6a19*) were analyzed. The results showed that *Slc6a19* levels were significantly reduced in fluoride-exposed Paneth cells (Figure 4B), indicating that abnormal absorption of leucine led to defective α-DEFENSIN secretion. Enrichment analysis based on KEGG gene sets of upregulated DEGs identified altered pathways in Paneth cells during fluoride exposure; compared with the Ctrl group, the top enriched pathways in the F group included those associated with pathogen invasion and intestinal inflammation, such as antigen processing and presentation, Epstein-Barr virus infection, human immunodeficiency virus 1 infection, intestinal immune network for IgA production, leishmaniasis, proteasome, and toxoplasmosis (Figure S1E). This suggests that the absence of defensins reduces the intestinal bacterial defense and induces bacterial invasion. Given that antimicrobial deficiency increases bacterial invasion and colonization of intestinal epithelial cells, the presence of luminal epithelial commensal bacteria were explored using fluorescence *in situ* hybridization (FISH). As expected, FISH revealed bacterial invasion of epithelial cells in fluoride-exposed mice (Figure 4D).

Intestinal mucus, secreted primarily by goblet cells, constitutes the first physical barrier of the gut, protecting the intestine from mechanical, biological, and chemical damage. The core components of the protective mucus barrier covering the intestinal epithelium include gel-forming mucin-2 (MUC2), mucus “shaper” calcium-activated chloride channel regulator 1 (CLCA1), and Fcγ binding protein (FCGBP), all involved in innate immune defense 45, 46. MUC2 immunostaining revealed that fluoride-exposed mice had thinner ileum mucus layers than the controls (Figure 4D). MUC2 is the main component of the outer mucus layer 47. Thus, focus was placed on the expression of mucus components in goblet cells of fluoride-exposed intestines and observed a slight increase in goblet-cell frequency in fluoride-exposed mice (Figure S1A and C). In contrast to the reduced mucus thickness observed in fluoride-exposed mice, ileal goblet cells in these mice displayed slightly increased mRNA levels of certain mucus-related genes (Figure 4E). These results indicate that the outer mucus layer deficiency in fluoride-exposed mice is not caused by transcriptional downregulation of mucus-associated genes.

Next, calcitonin gene-related peptide (CGRP) receptors, which are expressed in goblet cells and essential for maintaining the mucus barrier by driving goblet-cell emptying, were analyzed 48. CGRP receptors, particularly *RAMP1*, decreased in fluoride-exposed mice compared to controls (Figure 4G). In the absence of RAMP1, animals exhibited enhanced epithelial stress and susceptibility to pathogenic bacteria 48, 49. RAMP1 and MUC2 immunostaining supported the changes in *Ramp1* transcript levels (Figure 4H). Additionally, increased AB/PAS staining intensity in the fluoride-exposed ileum indicated that goblet cell emptying had been inhibited (Figure S1C). Transmembrane mucins (MUC1, MUC3, MUC4, MUC13, and MUC16) are localized on the apical surface of polarized epithelial cells forming a dense glycocalyx, separating commensal bacteria from epithelial cells 47. However, *MUC3a* levels were significantly reduced in the F group (Figure 4F), indicating that the barrier separating numerous commensal bacteria from epithelial cells was damaged 47.

Mucus biosynthesis involves complex processes and requires assembly in the endoplasmic reticulum (ER), including folding, carboxyterminal disulfide-mediated dimerization, initial N-glycosylation, and additional post-translational modifications in the Golgi apparatus such as O-glycosylation, as well as stabilization of the lumen by intramolecular crosslinking 46.

GO analyses of downregulated DEGs showed significant downregulation of pathways involved in molecular binding processes, the endomembrane system, and protein binding between the Ctrl and F groups (Figure S1D). These results suggest that fluoride exposure blocks mucus secretion.

Furthermore, the expression pattern of goblet cells showed gene signatures of Paneth markers, such as DEFA21 and LYZ1, indicating the presence of antibiotic function. This finding aligns with those reported by Wang *et al.* 50 and Jijon *et al.* 51 that goblet and Paneth cells are jointly responsible for the production and secretion of various antibacterial substances, including antimicrobial peptides, lectins, and lysozymes. scRNA-seq identified novel goblet-cell functions, enhancing our understanding of their contribution to intestinal homeostasis. A group of genes associated with antimicrobial defense (defensin family, *Lyz1*, *Zg16*, *Reg4*, *Lypd8*, *Ang4*, and *Pglyrp1*) displayed lower expression levels in fluoride-exposed goblet cells (Figure 4I).

DEFENSINS are broad-spectrum antimicrobial peptides effective against various microbes, including bacteria, fungi, and viruses 52. Reduced *Defensin* expression is associated with pediatric Crohn's disease 53. ZG16, a small lectin-like protein, limits gram-positive bacterial translocation into the mucus by binding to bacterial cell wall peptidoglycan 54. REG4 inhibits *Salmonella* invasion into intestinal epithelia by restraining its motility, growth, and proliferation, thereby ameliorating intestinal inflammation 55. Similarly, LYPD8 binds to the flagella of flagellated microbiota, preventing bacterial epithelial invasion 56. Goblet-cell loss and reduced LYPD8 are significantly associated with severe intestinal graft-versus-host disease 57. By contrast, antimicrobial peptide *Reg3g* levels were markedly upregulated in fluoride-exposed goblet cells (Figure 4I). Reactome enrichment analysis of downregulated DEGs indicated significant downregulation of pathways involving α-DEFENSINS, antimicrobial peptides, and the innate immune system in the F group (Figure 4J). LYZ1 staining using immunohistochemistry showed significantly decreased LYZ1 expression in goblet cells of the F group (Figure 4K).

### Fluoride exposure leads to lymphopenia of CD4^+^ T Cells and activates innate immune signaling in T cells

**Figure 5:**
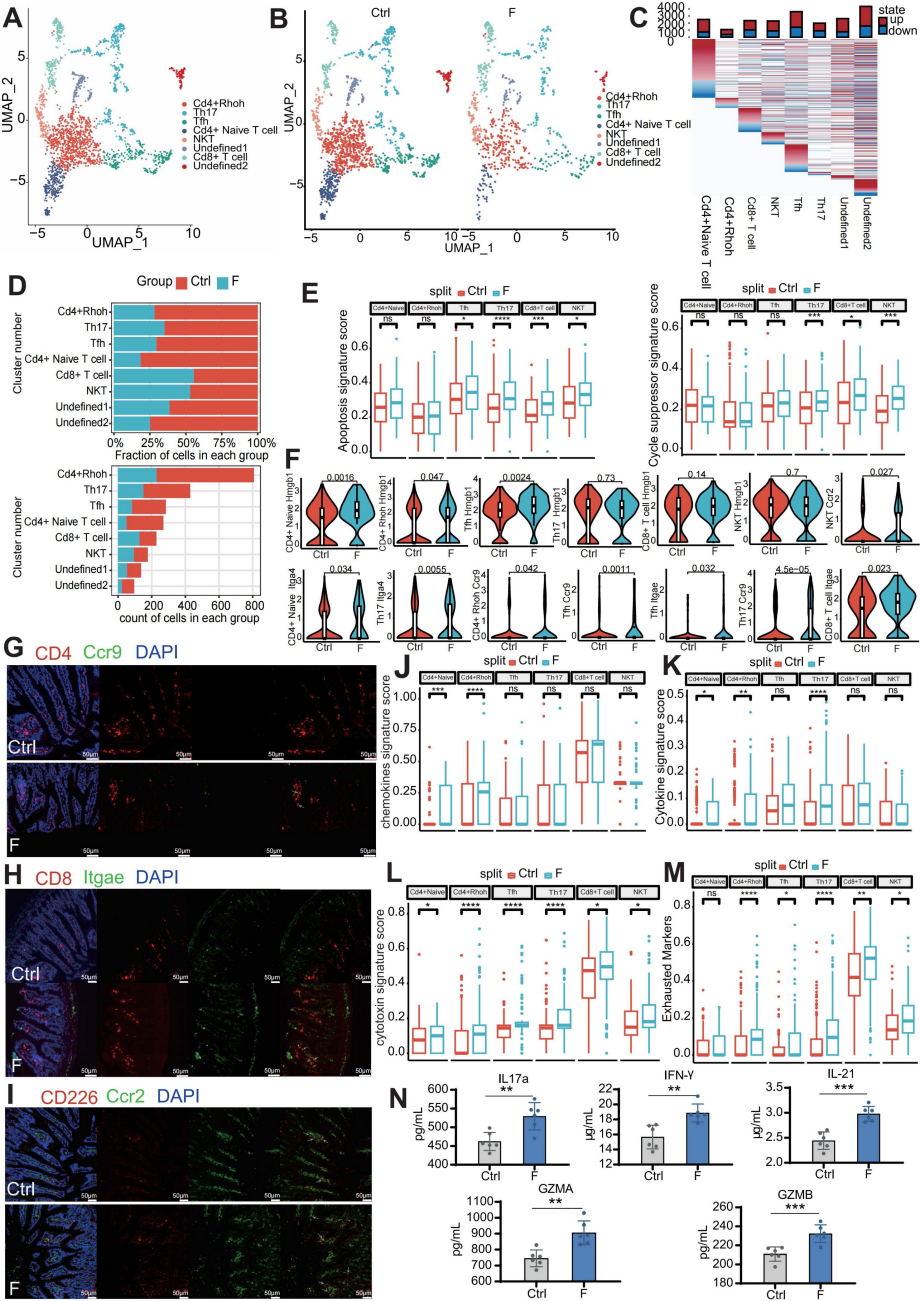
Fluoride exposure reduces clonal expansion of CD4^+^ T cells and activates innate immune signaling in T cells. (A) UMAP of sub-clustered T cells from unexposed and exposed mice. (B) UMAPs of T cells from unexposed (left) vs. exposed animals (right). (C) DEGs for each T-cell subtype; the top bars indicate the DEG count (red, upregulated; blue, downregulated). (D) Difference in the percentage of cells and cell count per cluster of T-cell subtypes (unexposed-exposed). (E) Box plot indicating the expression scores of apoptosis and cycle-suppressor genes in T-cell subtypes from the Ctrl and F samples. Significance was determined using the Wilcoxon test. (F) Violin plot showing the expression of *Hmgb1*, *Itag4*, *Itage*, *Ccr9*, and *Ccr2* in T cell subtypes from Ctrl and F samples, based on scRNA-seq. P-values were calculated using the Wilcoxon test. Representative immunofluorescence co-localization staining images for CD4 and CCR9 (G), CD8 and ITGAE (H), and CD226 and CCR2 (I) in ileum tissues from Ctrl- and fluoride-exposed mice (n = 3 biological replicates). White arrows indicate co-localization. Box plot indicating the expression scores of the chemokine (J), cytokine (K), cytotoxin (L), and exhausted (M) genes in T-cell subtypes from the Ctrl and fluoride samples. Significance was determined using the Wilcoxon test. (N) IL1, IFN-γ, IL-21, GZMA, and GZMB levels in the ileum from Ctrl and F groups were detected by ELISA. *, *p* < 0.05; **, *p* < 0.01; ***, *p* < 0.001; ****, *p* < 0.0001 were considered significant, and ns denotes non-significant differences.

Next, the immune cell composition and transcriptome were characterized to investigate the immune-system features in the small intestine of mice exposed to fluoride. First, unsupervised clustering of 2,426 T cells revealed eight subtypes based on distinct marker gene expression (Figure 5A and Figure S2A). These included four clusters of CD4^+^ T cells (CD4^+^ naive and CD4^+^ *Rhoh*, *Th17*, and *Tfh* T cells), one cluster of CD8^+^ T cells, one cluster of natural killer T (NKT) cells, and two clusters of undefined cells.

One undefined cluster (undefined2) was identified as B-cell contamination due to the expression of the B-cell marker *Cd79a* and was excluded from subsequent analysis. The other undefined cluster (undefined1) could not be identified using known marker genes. Frequency analysis of T cells revealed that CD4^+^ T cells (CD4^+^ naive and CD4^+^ *Rhoh*, *Th17*, and *Tfh* T cells) contributed to the reduction in the T-cell population in the F group compared to the Ctrl group, whereas CD8^+^ T and NKT cells were slightly increased in the F group (Figure 5B-D). Fluoride exposure altered the transcription of all T-cell subtypes (Figure 5C). In response to infection, CD4^+^ naive T cells proliferate and differentiate into various effector types, including conventional helper effector, regulatory, and follicular helper cells (TH1, TH2, TH17, TREGS, and TFH) 58, 59.

KEGG enrichment analysis suggested that the CD4^+^ *Rhoh* T cell population was most functionally related to the *Th1*, *Th2*, and *Th17* clusters, showing enrichment in TH1, TH2, and TH17 cell differentiation signaling pathways (Figure S1B). To determine the mechanism through which fluoride exposure results in T-cell lymphopenia, expression levels of apoptosis and cell-cycle suppressor genes were examined. T cells from the fluoride group showed higher modulation scores for apoptosis-related genes (*Tfh*, *Th17*, *Cd8^+^* T cells, and NKT cells) and cell-cycle suppressor genes (*Th17*, *Cd8^+^* T cells, and NKT cells) (Figure 5E and Table S2). Additionally, upregulation of *Hmgb1*, a significant damage-associated molecular marker, was observed in fluoride-exposed CD4^+^ naive T cells, CD4^+^ *Rhoh* T cells, and *Tfh* subsets (Figure 5F), as this molecule is released during necroptotic T-cell death to trigger severe inflammatory responses 60. By contrast, homing genes (*Itga4*, *Itgae*, and *Ccr9*) were upregulated in CD4^+^ naive T cells, CD4^+^ *Rhoh* T cells, and *Th17*, *Tfh*, and CD8^+^ T-cell subsets in the fluoride-exposed intestine, whereas *Ccr2* was enriched in the NKT subtype (Figure 5F). Next, multiplex immunofluorescence (mIF) was used to validate the observed variations in cell-type proportions from the scRNA-seq analysis. The mIF analysis showed a slight decrease in CD4^+^ T and a slight increase in CD8^+^ T and NKT cells (saturated with CD266) in fluoride-exposed intestines (Figure 5G-I). Consistent with our scRNA-seq findings, the fluorescence intensity of co-localization between CCR9 and CD4, ITGAE and CD8, and CCR2 and CD226 was found to be increased in fluoride-exposed intestines (Figure 5G-I). These findings suggest that the intestinal environment in the F group restricts the proliferation of T cell subsets, induces the loss (apoptosis/necroptosis) of T-cell subsets, and enhances the homing of TΗ17, CD8^+^ T cells, and NKT cells. The greater loss of CD4^+^ T cells compared with the number of homing CD4^+^ T cells explains the lower population of CD4^+^ T cells in the F group compared to that in the Ctrl group, whereas the opposite changes were observed in CD8^+^ T and NKT cell populations.

To further determine the unique transcriptional states of T-cell subsets in fluoride-exposed samples, expression scores of cytokines, chemokines, and cytotoxic and exhausted T-cell phenotypes were analyzed. The transcriptional profiles of CD4^+^ T cells in the F group showed activation features manifested by higher inflammatory signals, including elevated chemokine (Figure 5J) and cytokine signature scores (Figure 5K). This was particularly evident in the overexpression of inflammatory cytokines in CD4^+^ *Rhoh* (*Ifng*), *Tfh* (*Ifng*, *Il21*, and *Ilf3*) and *Th17* (*Ifng*, *Il16*, *Il17a*, and *Il22*), and chemokines in CD4^+^ naive T cells (*Ccl5* and *Cxcl10*), CD4^+^ *Rhoh* (*Ccl5*), *Tfh* (Ccl4), and *Th17* (*Ccl5*) (Table S3). The key signal factors IFN-γ (*Ifng*), IL17a (formally regarded as IL-17), and IL21 were detected by ELISA to assess the influence of activation and lymphopenia of the CD4^+^ T cells on the inflammatory state. These analyses confirmed that the levels of IL17a, IFN-γ, and IL21 in the F group were remarkably elevated compared with the Ctrl group (Figure 5N). Moreover, all CD4^+^ T-cell subsets in the F group displayed markedly higher cytotoxic signature scores (Figure 5L), indicating a differentiation toward cytotoxic CD4^+^ T cells (CD4 CTL) in the fluoride-exposed intestine 61. Most of these cytotoxic genes were significantly differentially expressed in CD4^+^ T-cell subsets (Figure S2E-H). CD8^+^ T cells were enriched in the cytotoxic genes *Granzyme A* (*Gzma*), *Gzmb*, *Gzmk*, *Srgn*, *Sytl2*, and *Prf1* (Figure S2C), similar to previously reported cytotoxic effector CD8^+^ T cells 62. Compared with the Ctrl group, *Gzma*, *Gzmb*, and *Sytl2* were significantly upregulated in fluoride-exposed CD8^+^ T cells, displaying higher cytotoxic signature scores (Figure 5L and Figure S3A) and suggesting that fluoride exposure induced an increase in cytotoxicity.

KEGG pathway analysis of upregulated genes indicated that the T-cell receptor signaling pathway and chemical carcinogenesis-reactive oxygen species were enhanced in CD8^+^ T cells of the fluoride group (Figure S2D). As previously reported 63, NKT cells displayed high expression of chemokine receptor genes *Cxcr3*, *CxcrR6*, and *Ccr5* (Figure S2C), suggesting a migratory phenotype.

Fluoride-exposed NKT cells exhibited higher cytotoxic signature scores due to enhanced *Gzma* and *Prf1* expression (Figure 5L and Figure S3A). Consistently, KEGG pathway analysis showed upregulated NKT cell-mediated cytotoxicity in fluoride-treated NKT cells (Figure S2K). Expression of pyroptosis-related genes *Gzma* and *Gzmb*, predominantly expressed in CD8^+^ T and NKT cells 64, was further assessed using ELISA. As shown in Figure 5N, the GZMA and GZMB levels were significantly increased in fluoride-exposed intestines compared to the Ctrl group, indicating an enhanced pyroptotic capability of CD8^+^ T and NKT cells. Almost all T-cell subsets in fluoride-exposed samples exhibited increased expression of exhausted signatures compared to those of the control samples (Figure 5M). Notably, the fluoride-exposed samples showed a remarkable downregulation of *α-Defensins* (*Defa21*, *Defa22*, *Defa24*, and *Defa30*) across all T-cell subsets compared to the Ctrl group (Figure S2E-J). These findings confirm that fluoride exposure upregulated inflammatory, cytotoxic, and exhausted signatures in CD4^+^, CD8^+^ T, and NKT cells.

### Fluoride exposure induces PC expansion by reprogramming transcription and gene regulation in germinal centers

**Figure 6:**
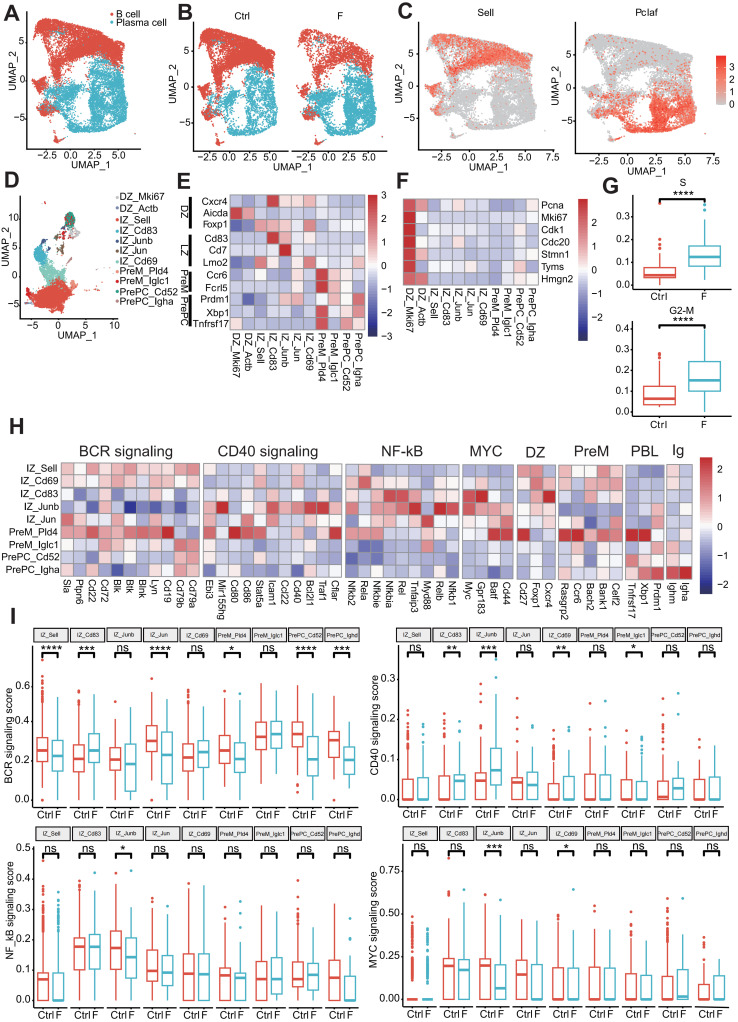
Fluoride exposure causes plasma cell expansion and germinal center B-cell reprogramming. (A) UMAP of sub-clustered B-lineage cells from unexposed and exposed mice. (B) UMAPs of unexposed (left) vs. exposed (right) samples. (C) UMAP projections are colored based on the expression z-score of two marker genes. (D) UMAP of sub-clustered B cells from unexposed and exposed mice. (E) Heat map displaying the relative expression as fold change (log_2_) of selected genes in the B-cell clusters identified in (D). (F) Heat map displaying the relative expression as fold change (log_2_) of cycle-related genes in the identified B cell clusters (D). (G) Box plot representing the gene expression score of the S and G2-M phases of the cell cycle in DZ-*Mki67* cells from control and fluoride samples. Significance was determined using the Wilcoxon test. (H) Heat map displaying the relative expression fold change (log_2_) of selected genes in IZ B cell clusters identified in B. (I) Box plot indicating the feature score of BCR signaling, CD40 signaling, NF-κβ activation, and MYC signaling in B-cell subtypes from control and fluoride samples. Significance was determined using the Wilcoxon test. *, *p* < 0.05; **, *p* < 0.01; ***, *p* < 0.001; ****, *p* < 0.0001 were considered significant, and ns denotes non-significant differences.

B-lineage cells are distributed in foci of highly organized B cells called gut-associated lymphoid tissue (GALT) and diffuse lymphoid tissues within the extensive intestinal lamina propria of the small intestine 64. GALT, which includes PPs and isolated lymphoid follicles, is characterized by ongoing germinal centers (GCs) 64, 65. GCs are divided into interconvertible compartments: the dark zone (DZ), characterized by intense B-cell proliferation and somatic hypermutation (SHM), and the light zone (LZ), characterized by affinity selection, with selection promoting interconversion between these compartments via signals from antigens and TFH 66, 67. Two signaling pathways promote positive selection of B cells in the LZ: the B-cell receptor (BCR) pathway, which directly recognizes antigen stimulation to obtain information on affinity selection, and the CD40 pathway, which indirectly receives affinity selection signals from B cells presenting antigens to TFH cells. Moreover, an intermediate transition region exists between DZ and LZ, defined as the intermediate zone (IZ). Differentiated PC populations migrate to GCs and enter the lamina propria 68. Based on marker gene expression and *Pclaf*, B-cell lineage cells were divided into B and effector B cells (PCs) (Figure 6A and C). Fluoride exposure significantly decreased the number of B and increased the number of PCs, indicating a promotion of the switch from B cells to PCs (Figure 6B and Figure S4A).

Unsupervised clustering of 11 different B-cell clusters, including two DZ clusters, five IZ clusters, two precursor memory B (PreM) clusters, and two precursor plasma (PrePC) clusters, was conducted based on distinct marker gene expression (Figure 6D and E). The expression of known marker genes for DZ cells (*Cxcr4*, *Aicda*, and *Foxp1*), PreM cells (*Ccr6* and *Fcrl5*), and PrePC cells (*Prdm1*, *Xbp1*, and *Tnfrsf17*) were highly restricted to their respective cluster (Figure 6E). IZ cells (IZ-*Sell*, IZ-*Cd83*, IZ-*Junb*, IZ-*Jun*, and IZ-*Cd69*) expressed gene features of both the DZ and LZ (*Cd83*, *Cd27*, and *Lmo2*) (Figure 6E). Fluoride exposure increased the diversity of GC cells, with the DZ-*Actb* cluster emerging as a specific cell type in fluoride-exposed tissue and serving as a disease marker (Figure 6D and Figure S4A). Consistent with previous reports 69, genes involved in the S-G2-M stages of the cell cycle were mainly enriched in the DZ compartments (DZ-*Mki67* and DZ-*Actb*) (Figure 6F), indicating that proliferative features were primarily associated with DZ GC B cells. Despite a reduction in B cells in the F group, the number of highly proliferating DZ B cells (DZ-*Mki67* and DZ-*Actb*) increased (Figure S4A). Consistent with the observations of Holmes *et al.* 69, PreM clusters (PreM-*Pld4* and PreM-*Iglc1*) were found to lack expression of S-G2-M genes (Figure 6F), indicating that GC B cells exit the cell cycle following differentiation into memory cells. In fluoride-exposed samples, DZ-*Mki67* cells displayed significantly higher proliferation signature scores (Figure 6G and Table S4).

KEGG pathway enrichment analysis of upregulated DEGs confirmed that the DZ-*Mki67* subtype in the fluoride group displayed significant involvement in proliferation and DNA repair pathways, including oxidative phosphorylation, cell cycle, DNA replication, base excision repair, nucleotide excision repair, and mismatch repair (Figure S4B), which are crucial for SHM 70.

Gene expression profiles associated with GC reactions were analyzed to clarify the dynamics of GC B-cell development. IZ-*Sell* was identified as an initial stage of activation in the LZ, termed “LZ-entry,” characterized by the enrichment of genes in the BCR but not in the CD40 signaling pathway (Figure 6H). This suggests a state ready for antigen contact but not for interaction with T cells. IZ-*Cd69* may represent subsequent LZ stages in which B cells show induction of genes of the CD40 signaling pathway (including *Cd40* and *Stat5a*) and NF-κB activation (including *Nfkbie*, *Rela*, and *Nfkb2*), in addition to BCR signaling (Figure 6H), indicating antigen capture. IZ-*Cd83*, IZ-*Junb*, and IZ-*Jun* displayed characteristics of strong T cell-mediated activation 69 due to the dominant features of the CD40 signaling pathway and NF-κB activation, along with enrichment of B-cell activation markers (*Batf*, *Cd44*, and *Gpr183*) and *Myc* expression (Figure 6H).

Notably, while some of these clusters maintained the expression of *Ighm*, whereas PreM-*Pld4* and PrePC-*Igha* expressed switched immunoglobulin receptors (Figure 6H). To determine changes in the affinity selection program, expression scores of BCR signaling, CD40 signaling, NF-κB activation, and MYC signaling features were analyzed. Compared with the normal intestine, the BCR-signaling-pathway feature score was significantly increased in the IZ-*Cd83* cluster and dramatically reduced in the IZ-*Sell*, IZ-*Jun*, Prem-*Pld4*, PrePC-*Cd52*, and PrePC-*Ighd* clusters in the fluoride-exposed intestine. The signature score of the CD40 signaling pathway was remarkably increased in IZ-*Cd83*, IZ-*Junb*, and IZ-*Cd69* and significantly reduced in PrePC-*Iglc1* of fluoride-exposed intestines compared with that of normal intestines. Additionally, the signature scores of NF-κB activation and/or MYC signaling were significantly reduced in IZ-*Junb* and IZ-*Cd69* clusters (Figure 6I). GC B cells recirculate between the DZ and LZ compartments or exit the GC by balancing the expression of gene signatures, including GC markers and an affinity selection program 69. Furthermore, the expression level of *Il21r* was significantly upregulated in IZ-*Sell* and IZ-*Cd69* cells in fluoride-exposed intestines compared with those of normal intestines (Figure S4C). Luo *et al.* 66 demonstrated that IL-21R signaling, in cooperation with CD40, drives the differentiation of GC B cells into PCs. These results indicate that fluoride exposure remodels the transcriptional program of GC B cells, promoting PC selection in GCs.

### Enrichment of highly differentiated PCs in fluoride-exposed intestines

**Figure 7:**
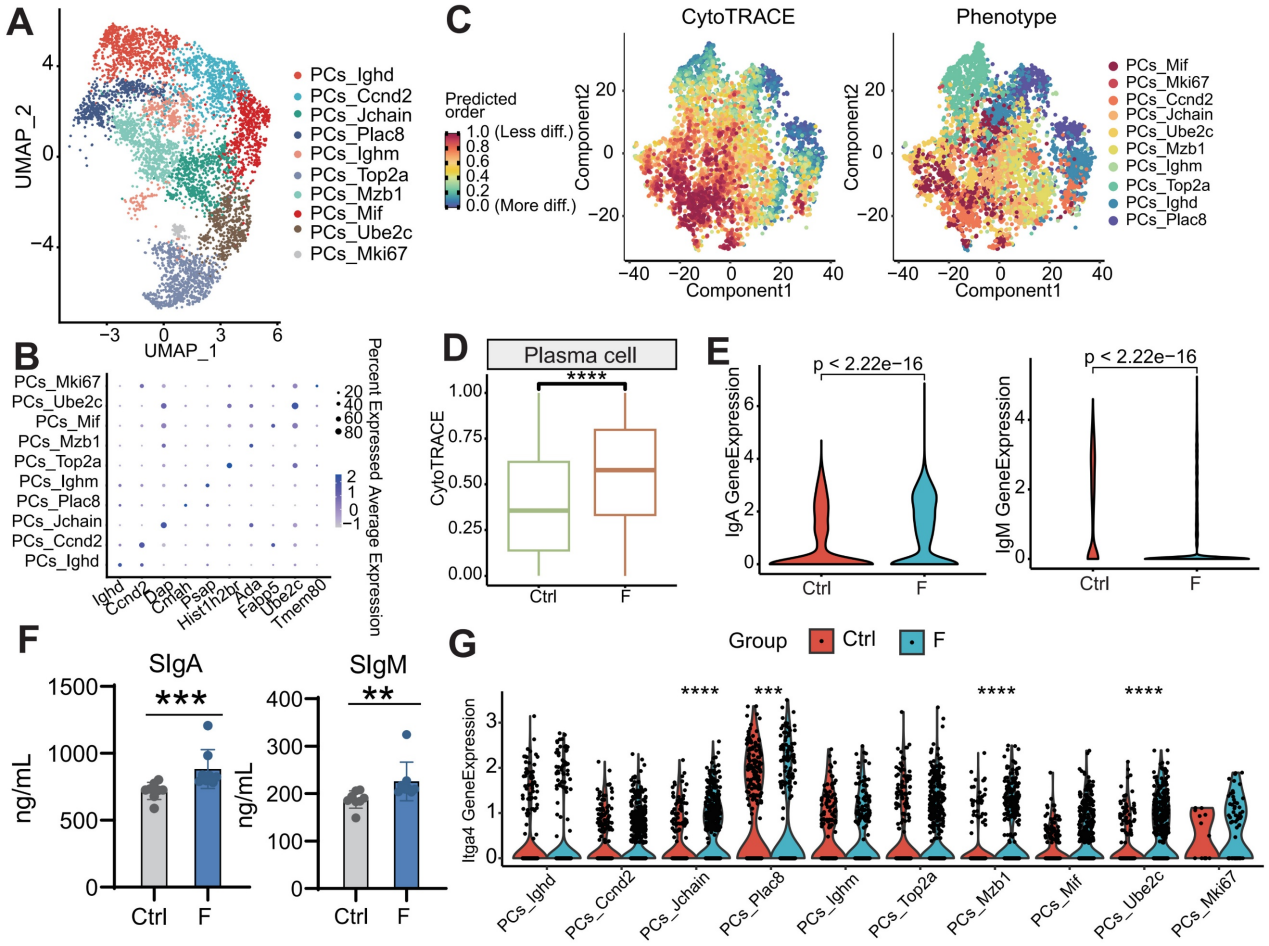
Differentiation status of PCs in fluoride-exposed ileum. (A) UMAP of sub-clustered PCs from unexposed and exposed mice. (B) Bubble chart showing the distribution and expression of marker genes in PC subsets from (A). (C) Visualization of the predicted differentiation using a CytoTRACE plot of PCs, showing the CytoTRACE score component with UMAP coordinates (left). Colors indicate the CytoTRACE score, ranging from 1 (red, lowest levels of differentiation) to 0 (blue, highest levels of differentiation). Feature plots showing PC subtype projections on the CytoTRACE plot (right). (D) Comparison of CytoTRACE scores between fluoride-exposed and normal intestines based on gene counts and the top 200 genes associated with the gene counts. (E) Gene expression levels of IgA and IgM in the F and Ctrl groups based on scRNA-seq. Significance was determined using the Wilcoxon test. (F) Contents of sIgA and sIgM in the ileal lumen of mice in the Ctrl and F groups as detected by ELISA. (G) Gene expression levels of Itga4 across all PC subtypes in the F and Ctrl groups based on scRNA-seq. Significance was determined using the Wilcoxon test. *, *p* < 0.05; **, *p* < 0.01; ***, *p* < 0.001; ****, *p* < 0.0001 were considered significant, and ns denotes non-significant differences.

Unsupervised clustering of PCs identified ten distinct subtypes based on marker gene expression (Figure 7A-B). The population of each subtype increased in the fluoride-exposed intestine (Figure S4J). CytoTRACE analysis, a trajectory reconstruction analysis that uses gene counts and expression to predict differentiation states in scRNA-seq datasets 66, was used to examine PC differentiation states. This analysis clarified the differentiation states of PC subtypes. The differentiated cells from the least (highest values) to the most (lowest values) differentiated were PCs-*Mif*, PCs-*Mki67*, PCs-*Ccnd2*, PCs-*Jchain*, PCs-*Ube2c*, PCs-*Ighm*, PCs-*Mzb1*, PCs-*Top2a*, PCs-*Ighd*, and PCs-*Plac8* (Figure 7C and Figure S4D). CytoTRACE values were significantly increased in the fluoride group (Figure 7D and Figure S4F), indicating a higher developmental potential for fluoride-exposed PCs than for normal PCs.

Genes positively correlated with high CytoTRACE values (indicating higher developmental potential) included *Ppia*, *H2afz*, *Ptma*, *Txn1*, *Ran*, *Rps8*, *Gapdh*, *Rps2*, *Npm1*, and *Rbm3*. Conversely, genes negatively correlated with high CytoTRACE values were *Fam43a*, *Mndal*, *Cd38*, *Btg1*, *S100a10*, *Gpr183*, *Ifi203*, *Pdcd4*, *Gm31243*, and *Ighd* (Figure S4E). IgA and IgM are key antibodies secreted by PCs for pathogen defense 66. According to our scRNA-seq analysis, the percentage of IgA^+^ and IgM^+^ PCs was lower in the F than in the Ctrl group (Figure S4G). Further investigation revealed that IgA and IgM expression was higher in fluoride-exposed samples compared with controls, as indicated by scRNA-seq (Figure 7E and Figure S4H-I). ELISA results confirmed that fecal levels of sIgA and sIgM were significantly increased in the F group (Figure 7F). Additionally, scRNA-seq analysis showed upregulation of the intestinal-homing gene *Itga4* in PCs-*Jchain*, PCs-*Plac8*, PCs-*Mzb1*, and PCs-*Ube2c* in fluoride-exposed samples (Figure 7G).

### Ligand-receptor analysis reveals intricate interactions between structural and immune cells

**Figure 8:**
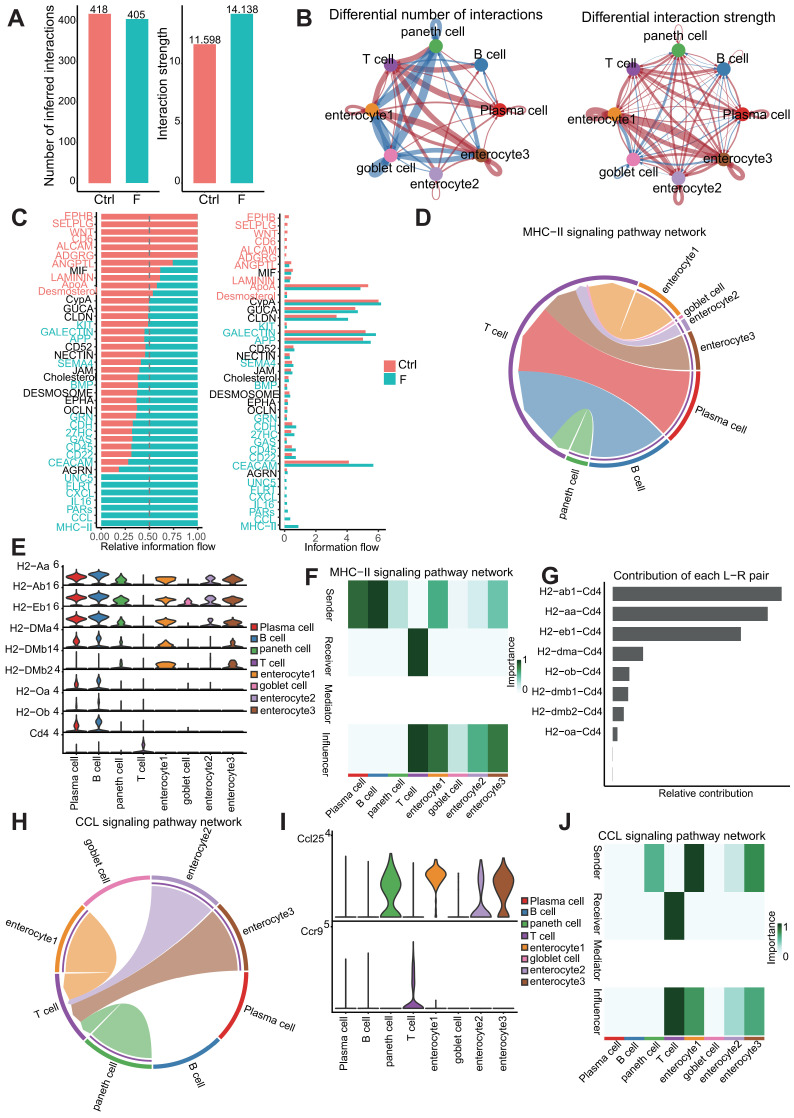
Ligand-receptor (L-R) analysis revealed an intestinal interactome network. (A) Number and strength of inferred interactions between the Ctrl and F group. (B) Circos plot showing the difference between the Ctrl and F group in terms of communication number and strength. Red indicates an increase in the F group, whereas blue indicates a decrease in the F group. (C) Overall information flow of each signaling pathway between the Ctrl and F groups. The top signaling pathways (*p* < 0.05) are labeled blue (enriched in the fluoride-exposed intestine) and red (enriched in the normal intestine). (D) Chord diagram showing the MHC-II signaling pathway network in the F group. (E) Expression levels of receptor and ligand genes of the MHC-II signaling pathway in the F group based on scRNA-seq. (F) Heat map of cells identified during the MHC-II signaling pathway in the F group. (G) Contribution of L-R pairs to the MHC-II signaling pathway. (H) Chord diagram showing the CCL signaling pathway network in the F group. (I) Expression levels of receptor and ligand genes of the CCL signaling pathway in the F group based on scRNA-seq. (J) Heat map of cells identified in the CCL signaling pathway in the F group.

Understanding the specific interactions between intestinal structural and immune cells in the intestinal microenvironment is crucial for elucidating the mechanisms underlying intestinal homeostasis and injury caused by fluoride exposure. Cell-communication pattern analysis among the dominant-intestinal-structure cells and immune cells was performed using CellChat. The number and strength of outgoing and incoming interactions were analyzed in each cluster in the Ctrl and F groups. A total of 418 and 405 Ligand-Receptor (L-R) interaction pairs were identified in samples from the Ctrl and F groups, respectively, with the F group showing enhanced interaction strength compared with that of the Ctrl group (Figure 8A).

Changes in the intercellular communication network during fluoride exposure primarily involved enterocyte clusters 1 and 3, and T cells (Figure 8B). Enterocyte clusters 1 and 3 displayed the highest signaling strength in both outgoing and incoming interactions under all conditions, indicating that enterocytes have the highest impact on other cells and could serve as a hub of cellular communication during intestinal physiology (Fig. S5A and B). Fluoride exposure resulted in global changes in interactions involving enterocyte clusters 1 and 3 and other cell types in both incoming and outgoing interactions, whereas T cells displayed increased incoming signaling strength and numbers (Figure 8B).

Signaling pathways involved in cell-cell-communication differences in the ileal microenvironment after fluoride exposure were further investigated. Figure 8C indicates that the upregulated signaling pathways in the fluoride-exposed ileum included KIT, GALECTIN, APP, SEMA4, BMP, GRN, CDH, 27HC, GAS, CD45, CD22, CEACAM, UNC5, FLRT, CXCL, IL16, PARs, CCL, and MHC-II. Notably, the CXCL, IL16, CCL, MHC-II, and GALECTIN pathways were active in T-cell signaling after fluoride exposure (Figure S5C-D), whereas GALECTIN, APP, CD22, and CD45 were involved in fluoride-exposed B and PCs signaling patterns (Figure S5C-D).

Enhanced outgoing signaling patterns of immune cells during fluoride-induced progression involved the MHC-II, CD22, CD45, PARs, and IL16 pathways, which belong to the adaptive immunity signaling pathway (Figure 8C and Figure S5C-D). Among these pathways, MHC-II and CCL, two important proinflammatory signaling pathways, exhibited the most significant increases in information flow in the fluoride-exposed intestine (Figure 8C). The chord diagram and heat map of cell identification indicated that the MHC-II signaling network in the intestine was simple, involving PCs and B cells as the sources of MHC-II ligands in immune cells, enterocytes (enterocyte clusters 1-3), goblet, and Paneth cells as the sources of MHC-II ligands in structural cells (Figure 8D and F). The MHC-II signal receivers were T cells (Figure 8D and F). The significantly enhanced H2-AA/CD4, H2-EB1/CD4, and H2-AB1/CD4 interactions between T cells and enterocyte cluster 1-3/B cells were the most significant contributors to the MHC-II signaling pathway under fluoride exposure (Figure 8G and Figure S5K). CCR9/CCL25 signaling is crucial for immune cell homing in the intestine 71. Notably, CCL signaling was also among the most significantly upregulated in fluoride-exposed intestines (Figure 8C). A chord diagram and cell heat map indicated that the sources of CCL ligand included enterocytes (enterocyte clusters 1-3) and Paneth cells, with T cells as the primary receivers (Figure 8H-J). The significantly enhanced *Ccl25*/*Ccr9* interactions between T cells and enterocyte clusters 1-3/Paneth cells contributed to the enhancement of the CCL signaling pathway following fluoride exposure (Figure 8G and Figure S5K).

Moreover, *Lgasl9* (*Galectin-9*)/*Cd45*, belonging to galectin signaling, was the dominant L-R pair between T cells and their ligands (enterocyte clusters 1 and 3, goblet, and Paneth cells), with communication enhanced by fluoride exposure (Figure S5K). Further analysis of T-cell crosstalk as a single sender revealed increased CYPA interactions with enterocyte clusters 1 and 3 via the *Pipa*/*Bsg* L-R pair (CYPA signaling), increased PARs interactions with goblet cells via the *Gzma*/*Pard3* L-R pair, and increased CD22 interactions with PCs via the *Ptprc*/*Cd22* and *Cd52*/*Siglecg* (CD52 signaling) L-R pairs. Moreover, enhanced structural cell crosstalk was concentrated at the CEACAM interaction via the *Ceacam1*/*Ceacam1* L-R pair (Figure S5E-I).

### Enterocyte MHC class II signaling mediates immune activation

**Figure 9:**
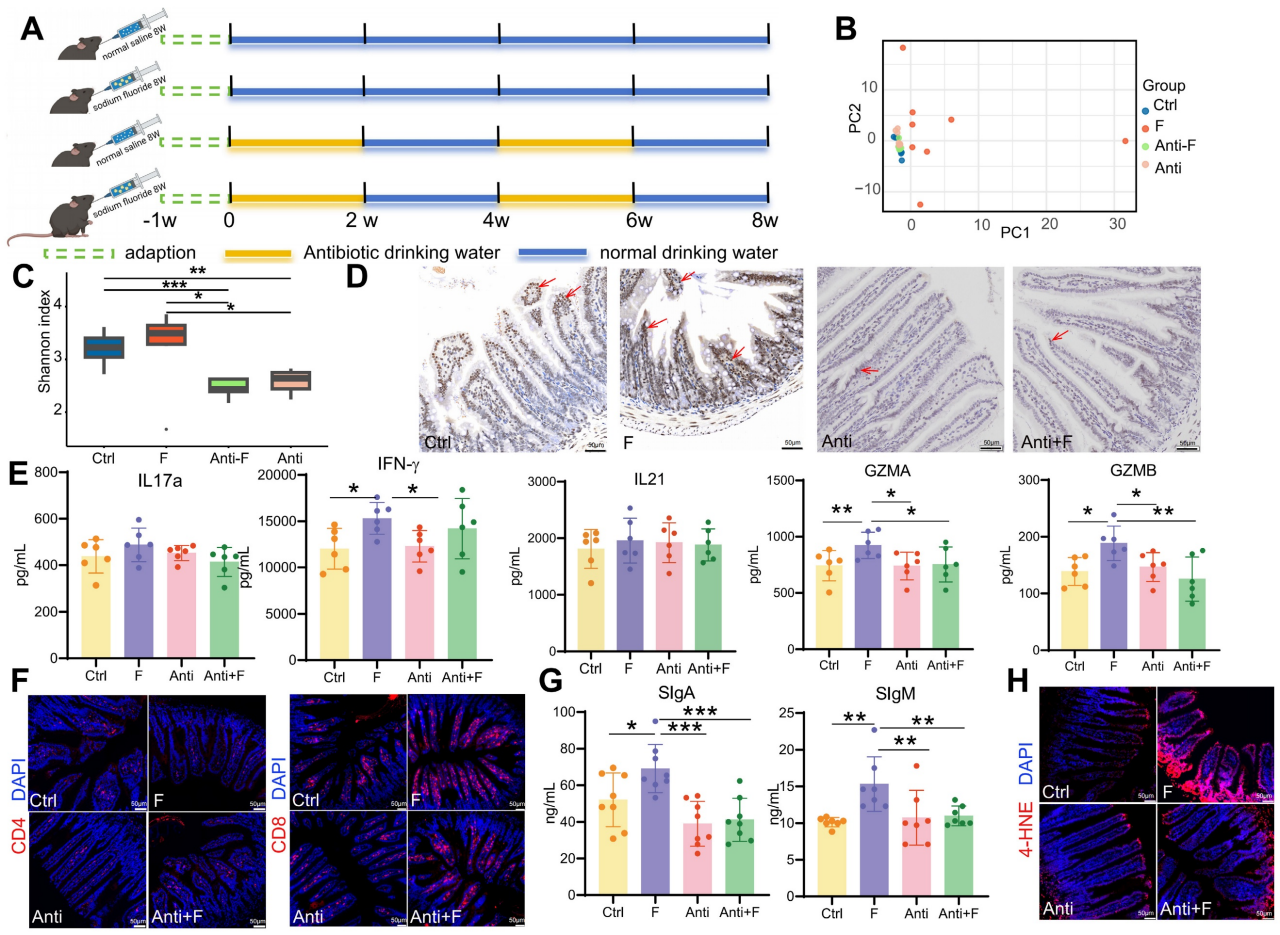
Dampening MHC class II expression in enterocytes inhibited immune activation. (A) Timeline schematic for experimental design. (B) Principal components analysis of Euclidean distance among groups. (C) Gut microbiome community richness (Shannon index) in each group. Significance was determined using the Wilcoxon test. (D) Immunohistochemistry staining of H2-AB in the mouse ileum (n = 3 biological replicates). The brown staining indicated by the red arrow represents positive expression of H2-AB. (E) IL-17a, IFN-γ, IL-21, GZMA, and GZMB levels in the ileum from Ctrl and F groups were detected by ELISA. (F) Immunofluorescence staining of CD4 and CD8 in the mouse ileum (n = 3 biological replicates). (G) Contents of sIgA and sIgM in the ileal lumen of mice in the Ctrl and F groups as detected by ELISA. (H) Immunofluorescence staining of 4-HNE in the mouse ileum (n = 3 biological replicates). *, *p* < 0.05; **, *p* < 0.01; ***, *p* < 0.001; ****, *p* < 0.0001 were considered significant, and ns denotes non-significant difference.

The intestinal microbiome plays a significant role in regulating intestinal immunity. Fluoride exposure was reported to massively alter the intestinal microbiome and increase bacterial diversity and richness 33. Similarly, fluoride exposure was observed to induced a significant separation of the gut microbiome between the control and fluoride group (Figure 9B), and resulted in a slightly increased bacterial richness in the fluoride group compared to the control group (Figure 9C). Beyaz *et al.* 72 confirmed that the administration of broad-spectrum antibiotics can effectively inhibit the expression of epithelial MHC class II by eliminating the intestinal flora. To corroborate the role of the intestinal microbiome on MHC class II expression in enterocytes, mice were treated with broad-spectrum antibiotics (Figure 9A), which ablated the bacterial richness and without influenced the community composition in the Anti group compared to the Ctrl group (Figure 9B-C). Moreover, antibiotic treatment inhibited the increase of bacterial richness (Figure 9C) and reversed the changes of community composition (Figure 9B) in fluoride exposed-mice. Concurrent with reducing bacterial richness, antibiotic treatment decreased MHC class II gene expression in enterocytes of normal mice and inhibited its upregulation in fluoride-exposed mice (Figure 9D). The variation trend of CD4^+^ and CD8^+^ T cell numbers induced by fluoride exposure were not changed by antibiotic treatment (Figure 9F). To decipher whether MHC class II upregulation affects the activation of CD4^+^ and CD8^+^ T cells *in vivo*, the alteration of cytokines and cytotoxins were further detected. The IL17a, IFN-γ, IL21, GZMA and GZMB levels exhibited no significant alteration in the intestines of mice treated with antibiotics alone compared with that of control mice (Figure 9E), which indicated that antibiotic treatment do not influence on T cells. By contrast, antibiotic treatment significantly inhibited the increase of intestinal IFN-γ, GZMA and GZMB in fluoride-exposed mice (Figure 9E), indicating MHC class II-mediated activation of T cells. Moreover, the antibiotic treatment also reversed the upregulation of intestinal sIgA and sIgM (Figure 9G) and lipid peroxidation (Figure 9H) induced by fluoride exposure, whereas it did not influence these indicators in mice treated with antibiotics alone compared with control mice.

## Discussion

Exposure to fluoride over a lifetime can lead to fluorosis, a silent geogenic disease affecting approximately 200 million people in 28 developed and developing countries. Research has demonstrated that excessive fluoride intake can cause intestinal inflammation and oxidative stress, but the underlying mechanism remains unclear. For the first time, the gene expression profiles of fluoride-exposed intestines were investigated via scRNA-seq to determine the cellular and molecular changes associated with fluoride exposure.

Intestinal epithelial cells are protected by mucus and a surface coating consisting of a dense glycocalyx 47. Mucins secreted by specialized goblet cells are the main components of both the mucus and the glycocalyx. Fluoride exposure was found to reduced the mucus thickness, accompanied by a slight increase in mucus gene expression. We explored the CGRP-RAMP1 signaling pathway, a specific signaling pathway within goblet cells required for emptying 48. scRNA-seq and immunofluorescence co-localization verified that the CGRP receptor *Ramp1* expression was significantly reduced in goblet cells, both at the mRNA and protein levels. Moreover, fluoride-exposed ilea lacked a surface-coated glycocalyx owing to the downregulation of transmembrane mucin *MUC3a* expression. Apart from the mucus barrier, fluoride exposure also changed the chemical barrier consisting of goblet and Paneth cells with reduced expression of *α-Defensin*, *Lyz1*, *Zg16*, *Reg4*, *Lypd8*, *Ang4*, and *Pglyrp1*. DEFENSINS are involved in many physiological processes, including direct antimicrobial functions, gut microbiota regulation, mucosal barrier maintenance, and immunomodulation 73, 74. α-DEFENSIN levels are negatively correlated with Bacteroides abundance 75 and play a critical role in the defense against *Salmonella typhimurium* 44, 76. Similarly, Wang *et al.* 55 verified that REG4 specifically binds to the *Salmonella* flagella to prevent them from invading intestinal epithelial cells and suppressing the host's inflammatory response. The *α-Defensin* and *Reg4* deficit may be associated with *Salmonella* infection pathway enrichment in enterocyte clusters 1-3. ZG16 is essential for normal host-bacteria symbiosis because it does not kill commensal bacteria but limits bacterial penetration into the host. Bergström *et al.* 54 discovered that in *Zg16^-/-^* mucus, gram-positive bacteria can migrate to systemic tissues due to increased bacterial motility within the mucus. Alterations in these antimicrobial substances may underlie gut microbiota disorders in the ileum and contribute to increased intestinal flora diversity, as indicated in our previous study 33. Moreover, damage to the mucus and glycocalyx, along with reduced antibacterial components, increases the likelihood of bacterial invasion into the intestinal epithelium.

Enterocytes, the most abundant differentiated intestinal epithelial cells, constitute a physical and biochemical barrier, absorb nutrients, produce antimicrobial peptides, and act as immunomodulators 77. Further investigation confirmed that fluoride exposure disrupted enterocyte-specific trans**-**differentiation, inhibiting the maturation of middle transition state enterocytes (enterocyte cluster 3) in the fluoride-exposed group, which impaired the transfer of intermediate cells to the top of the villi. This was evidenced by decreased expression of maturation marker genes in middle transition state cells and defects at the villus tips. Additionally, the increased number of enterocyte cluster 3 and decreased enterocyte cluster 2 cells in the F group confirmed that high-fluoride exposure inhibited the differentiation from enterocyte cluster 3 to 2.

Consistent with the findings of Moor *et al.* 36, the expression pattern of functional genes along the villi axis represents a transition from an antibacterial gene program at the bottom to a nutrient absorption program at the top of the villi. The transporters of key nutrient families (carbohydrates, lipids, vitamins, peptides, and amino acids) are suppressed in response to fluoride exposure. Our results also suggest that structural cell types influence immune responses. Accompanying the bacterial invasion of intestinal enterocytes in the fluoride-exposed intestine, the antimicrobial program of the enterocytes at the bottom of the villi was activated. For example, fluoride exposure upregulated the expressions of antimicrobial peptides (*Reg3b* and *Reg3g*), inflammatory protein *Saa1*, bacterial pathogen adherence protein *Clec2e*, and immune-related MHC class II genes (*H2-Aa*, *H2-Eb1*, *H2-Ab1*, and *H2-DMb1*) in these enterocytes. In conclusion, fluoride disrupted enterocyte trans**-**differentiation, causing apical villi defects, absorption dysfunction, and activation of the antimicrobial program in enterocytes at the bottom of the villi.

In this study, a distinct T-cell composition in fluoride-exposed intestines was identified, characterized by the exclusion of CD4^+^ T cells and enrichment of CD8^+^ T and NKT cells. Despite the reduction in CD4^+^ T-cell percentages, UCell analysis demonstrated that CD4^+^ T cells in the F-group exhibited activated transcriptional characteristics, with significantly elevated expression of chemokines, cytokines, and cytotoxins. Numerous inflammatory factors, including *Ifng*, *Il21*, *Ilf3*, *Il16*, *Il17a*, and *Il22*, were upregulated at the mRNA level in effector CD4^+^ T cells. ELISA analysis for several critical inflammatory factors verified that the decrease in CD4^+^ T cells did not result in immunosuppression. By contrast, the activated state of CD4^+^ T cells aggravated the intestinal inflammation, displaying remarkably increased IFN-γ and IL17a levels. Similarly, Zhu *et al.* 78 found that fluoride exposure increased the IL17a and IL22 levels in the colon, disrupting colonic homeostasis. Our previous study also confirmed significant increases in IFN-γ at both the mRNA and protein levels in the ileum 79. IL17a signaling was reported to be involved in myeloid cell recruitment 80. IFN-γ can directly induce apoptosis in intestinal epithelial cells and promote antigen presentation to T cells through the MHC class I and class II pathways 81. TFH-derived-IL-21, a B-cell differentiation factor, is involved in the early emergence of plasmablasts at the GC-T cell interface 82. The lymphopenia of CD4^+^ T cells may be attributed to fluoride exposure restricting T subsets proliferation and promotes apoptosis. The increased homing number of CD4^+^ T cells due to fluoride exposure could not compensate for the lost cells, based on UCell analysis of apoptosis-related and cycle-suppressor gene signatures, as well as homing-associated gene expression. The GALECTIN-binding T-cell surface glycoprotein receptor CD45 suppresses T-cell proliferation 83 and induces T cell death 84. The enhanced GALECTIN interaction between T cells and structural cells, particularly enterocytes clusters 1 and 3, via the LGASL9 (also called GALECTIN-9)/CD45 L-R pair in fluoride-exposed intestines, explains the restricted proliferation and increased apoptosis of T cells.

Wang *et al.* 85 found reduced percentages of CD3^+^, CD4^+^, and CD8^+^ T lymphocytes in fluoride-exposed spleens. The spleen is an important peripheral immune organ, and the exhaustion of T cells in the spleen is likely to cause further inhibition of intestinal-homing T cells, eventually leading to intestinal immunosuppression. In cellular immunity, CTLs and NK cells rely on perforin to deliver serine protease granzymes to target cells and kill them 86. CD8^+^ T cells exhibited CTL characteristics due to the high expression of *Gzma*, *Gzmb*, and *Gzmk*. Fluoride exposure significantly upregulated the expression of cytotoxic molecules, including *Gzma*, *Gzmb*, and *Sytl2* in CD8^+^ T cells. The expression of the cytotoxic molecules GZMA and PRF1 also increased in fluoride-exposed NKT cells.

Granzymes, particularly the two most abundant members (GZMA and GZMB), have been a major focus. GZMA from cytotoxic lymphocytes targets *GasderminB^+^* (GSDMB) cells, cleaving and activating GSDMB to induce target cell pyroptosis 86. IFN-γ upregulates *Gsdmb* expression and promotes GZMA-induced targeted cell pyroptosis 86. *Gsdmb* is highly expressed in the digestive tract epithelia, highlighting the importance of GZMA/GSDMB-mediated immunity in the digestive system 86. GZMB induces target cell apoptosis by activating caspase-3 or its substrates 86, 87. In summary, cytotoxic molecules play significant roles in immunity against fluoride exposure-induced infections.

The infiltration characteristics of B-lineage cells into the intestine during fluoride exposure remain poorly understood. Intestinal B-cell responses are initiated in organized GALT and characterized by ongoing GC reactions 65, 87. GC B cells generate memory or antibody-producing B cell (PCs) processes that involve SHM editing of the BCR, antibody affinity maturation, and class switch recombination, which are sources of high-affinity antibodies against pathogens 65. Fluoride exposure significantly increased the proliferation of DZ-*Mki67* cells. Proliferation within the DZ B cells is the basis of BCR clonal diversity via SHM 65. Pathways critical for SMH, such as oxidative phosphorylation, cell cycle, DNA replication, base excision repair, nucleotide excision repair, and mismatch repair, were enriched in fluoride-exposed DZ-*Mki67* cells, indicating that fluoride exposure enhanced SHM activity. DZ B cells typically migrate from DZ to LZ in response to activation and at the expense of their proliferative capacities 65, 88. Using scRNA-seq, a fluoride-specific DZ-*Actb* subtype that was prepared to migrate to the LZ from the DZ was identified, characterized by lower expression of cell cycle and DZ markers. Affinity selection of GC B cells in the LZ requires B-T-cell interactions that trigger signaling pathways driving DZ re-entry or differentiation into post-GC memory B cells and PCs. UCell analysis indicated that fluoride exposure changed the scores of BCR signaling, CD40 signaling, NF-κB activation, and MYC signaling in most IZ subsets, reprogramming GC transcription. In addition to the increased CD40 signaling score, *Il21r* expression was significantly upregulated in IZ-*Cd69* cells in fluoride-exposed intestines. Luo *et al.* 66 demonstrated that IL-21R and CD40 synergistically drive GC B-cell differentiation into PC. These results confirm that compared with normal intestines, fluoride-exposed intestines exhibit a significant skewing of B cells toward effector B cells (PC), manifested as a drastic decrease in the proportion of B cells and a substantial increase in the proportion of PCs.

Using CytoTRACE, we predicted a higher developmental potential for fluoride-exposed PCs compared with normal PCs. Luo *et al.* 89 found that high-fluoride exposure decreased the number of IgA^+^ positive cells in the lamina propria and reduced the levels of IgA and IgM in the ileum mucosa. By contrast, our study demonstrated that fluoride exposure decreased the percentage of IgA^+^ and IgM^+^ cells among the total PCs while promoting IgA and IgM secretion in the ileum based on scRNA-seq profile characteristics and ELISA detection of sIgA and sIgM in feces. In summary, extensive remodeling of B-lineage cells in the fluoride-exposed ileum was demonstrated. The SHM of DZ B cells and affinity selection of IZ B cells were enhanced by fluoride exposure, resulting in a shift from B cells to PCs, which manifested as decreased B cell and increased PC fractions with higher developmental potential.

In this study, CellChat analysis verified that enterocytes are the central communication hubs, emphasizing the importance of two proinflammatory signaling pathways (CCL and MHC-II) in cell communication. T cells were the only receivers of the two major upregulated L-R signaling pathways (MHC-II and CCL) during fluoride exposure. Although MHC class II function and expression are canonically considered restricted to professional antigen-presenting cells, several studies have demonstrated that intestinal epithelial cells (IECs) express high levels of MHC class II and can capture, process, and present antigens to T cells 72, 90. Both human and murine studies demonstrated that elevated IEC MHC-II expression allows IECs to activate immune responses in celiac and inflammatory bowel disease (IBD) 91. In consideration of the key role of the intestinal microbiome in the upregulation of IEC MHC II expression 72 and bacterial invasion in the fluoride-exposed intestine, broad-spectrum antibiotics were used to clear the intestinal flora and found that fluoride-induced elevation of IEC MHC II expression was reversed, which indicated that epithelial MHC class II expression in fluoride-exposed mice depended on the intestinal microbiome. Jamwal *et al.* 91 demonstrated that MHC-II in IECs influences the severity of T-cell-induced and infectious colitis in mice. Specifically, the loss of MHC-II in these cells alleviated the severity of T-cell transfer-induced colitis accompanied by a reduced luminal concentration of sIgA 91. Similarly, inhibiting the expression of MHC-II in enterocytes of fluoride-exposed mice alleviated the activation of T cells, manifested by the reduced ileal IL17a, IFN-γ, GZMA, and GZMB levels. L-R analysis demonstrated enhanced crosstalk between T and B cells via antigen presentation through BCR signaling (H2-AA/CD4, H2-EB1/CD4, and H2-AB1/CD4), which is required for GC formation and stronger positive selection for generating gut-homing PCs producing high affinity IgG and IgA antibodies 65, 92. Accompanying the inhibition of T-cell activity, the fluoride-induced high levels of ileal sIgA and sIgM were reduced. Accordingly, these results confirm that that fluoride-induced elevation of MHC-II expression in villus enterocytes was the core of a downstream series of immunoreactions. Fluoride exposure upregulates the CCL interaction between T cells and enterocytes via the CCL25/CCR9 L-R pair, promoting the arrival of T cells at injury sites, as well as through immune-related components of MHC class II genes presenting antigens to T cells that activate downstream signaling.

Though the impact and mechanisms of lifelong exposure to naturally occurring moderate-to-high-fluoride drinking water on intestinal function have been, to some extent, demonstrated by single-cell RNA sequencing, there are still some limitations to this study. First, the gene expression profiles detected in the ileum cannot be extrapolated to the entire intestinal landscape, as the analysis was restricted to this specific intestinal segment. Second, the tissues were collected and analyzed at a single time point (after 56 weeks of treatment), which could not represent the temporal changes and dynamics of biological responses to high-fluoride exposure. Last, a single mouse strain (C57BL/6J) was used in this study. As different strains might have different responses to high-fluoride exposure, the generalizability of the findings is somewhat limited.

## Conclusions

In this study, we clarified the cellular and molecular signatures of fluoride-exposed intestinal epithelial and immune cells and elucidated the mechanisms underlying intestinal injury. Fluoride exposure destroyed the mucus barrier, allowing bacteria to invade the intestinal epithelium. This invasion inhibited nutrient transporter expression, leading to dysfunctional absorption by intestinal villi, and induced activation of the antimicrobial response in enterocytes. Activated enterocytes presented antigens to T cells through L-R pairs of the MHC-II signaling pathway, causing downstream reactions, including upregulation of proinflammatory factors and cytotoxic molecules and remodeling of B-lineage cells. Our findings underscore the importance of monitoring and regulating fluoride levels in drinking water and other sources to prevent fluorosis and related intestinal damage. Future studies could deploy multiple time-point analyses to address the limitations of a single time-point analysis. Furthermore, additional studies can involve different mouse strains to broaden the generalizability of our study findings.

## Supplementary Material

Supplementary figures.

Supplementary tables.

## Abbreviations

sIgA: secretory IgA
Th17: T helper 17
scRNA-seq: Single-cell RNA sequencing
PDE: phosphodiesterase
DPBS: Dulbecco's phosphate-buffered saline
FBS: fetal bovine serum
GEMs: Gel Beads-in-emulsion
RT: reverse transcription
PCA: principal component analysis
UMAP: Uniform Manifold Approximation and Projection
DEGs: Differentially expressed genes
GO: Gene Ontology
KEGG: Kyoto Encyclopedia of Genes and Genomes
DEPC: paraformaldehyde
ELISA: enzyme-linked immunosorbent assay
sIgM: secretory IgM
M cells: microfold cells
PP: Peyer's patches
HE: Hematoxylin-eosin
FISH: fluorescence *in situ* hybridization
PCs: plasma cells
MUC2: mucin-2
CLCA1: calcium-activated chloride channel regulator 1
FCGBP: Fcγ binding protein
CGRP: calcitonin gene-related peptide
ER: endoplasmic reticulum
mIF: multiplex immunofluorescence
CD4 CTL: cytotoxic CD4^+^ T cells
Gzma: Granzyme A
GALT: gut-associated lymphoid tissue
GCs: germinal centers
DZ: dark zone
SHM: somatic hypermutation
LZ: light zone
BCR: B-cell receptor
IZ: intermediate zone
PreM: precursor memory B
PrePC: precursor plasma
L-R: Ligand-Receptor
GSDMB: GasderminB
