# Supplementary material for: Single-cell RNA sequencing uncovers intestinal immune alterations and cellular diversity from chronic fluoride exposure in mice
Source: Theranostics. 2025 Jun 18;15(15):7242–69. doi: 10.7150/thno.116567 (PMC12315694; doi:10.7150/thno.116567)
Supplement: Supplementary file 1 — Supplementary figures. [file thnov15p7242s1.pdf]

Single-cell RNA sequencing uncovers intestinal immune alterations and cellular diversity from chronic fluoride exposure in mice

Supplementary Information

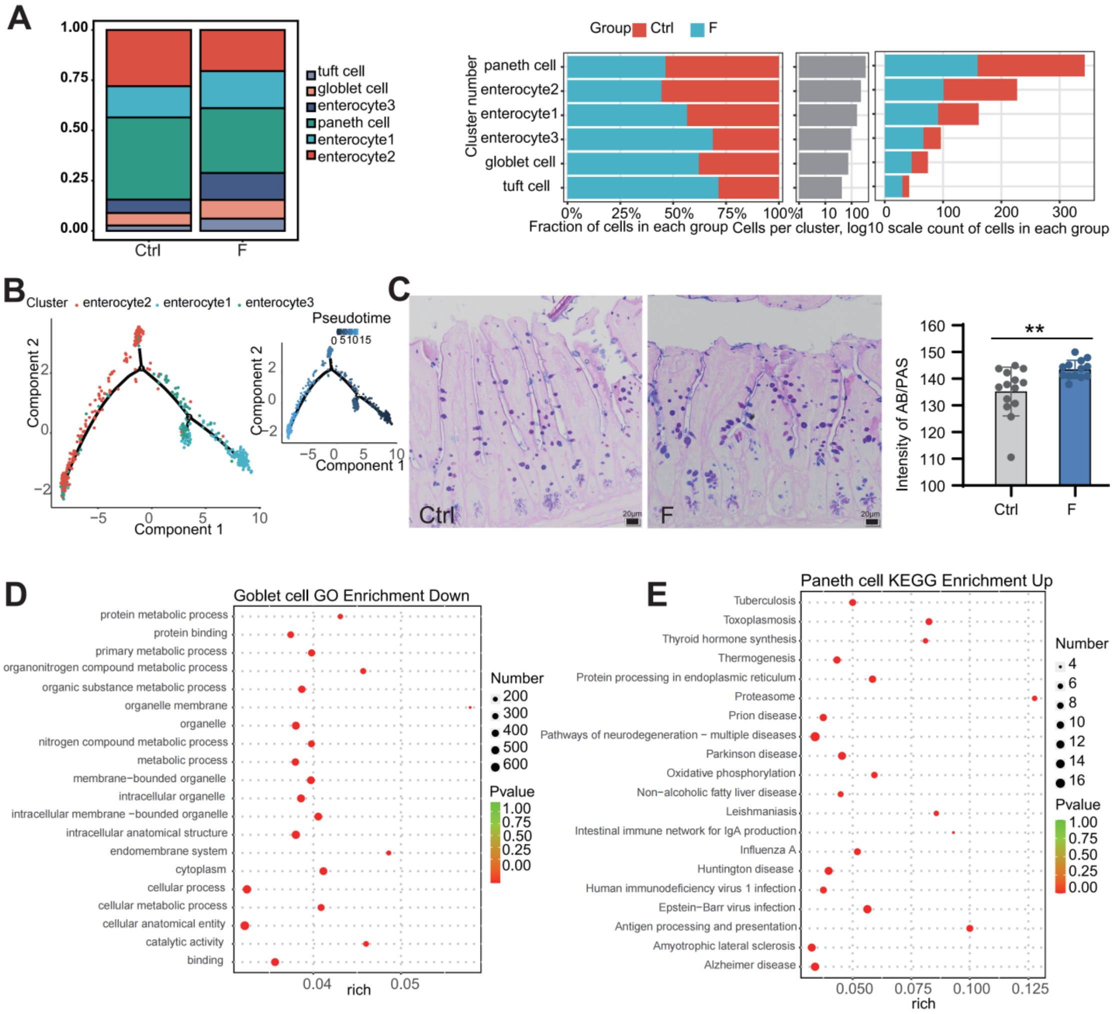

Figure S1 The influences of fluoride exposure on epithelial subsets, related to Figure 2-4. (A) Stacked bar chart of ratio of epiththelial cell subtypes ((unexposed-exposed)). (B) Mapping of the enterocyte clusters on the pseudo-time plot. Pseudotime of the enterocyte cluster calculates by Monocle 2. The projection is colored by pseudo-time. (C) AB/PAS staining of ileal tissues from the Ctrl and F groups. The glycogen and neutral mucous substances in the tissue are red, the acidic mucous substances are blue, and the mixed mucous substances are bluish-purple or purple-blue. Quantification of ileal goblet cell mucus granule emptying by measurement of AB/PAS staining intensity in mice from (C) (n = 4–5 representative visions/mouse and 3 mice/ group).

(D) Go enrichment analysis of goblet cell based on the down-regulated DEGs. (E) KEGG enrichment analysis of paneth cell based on the up-regulated DEGs.

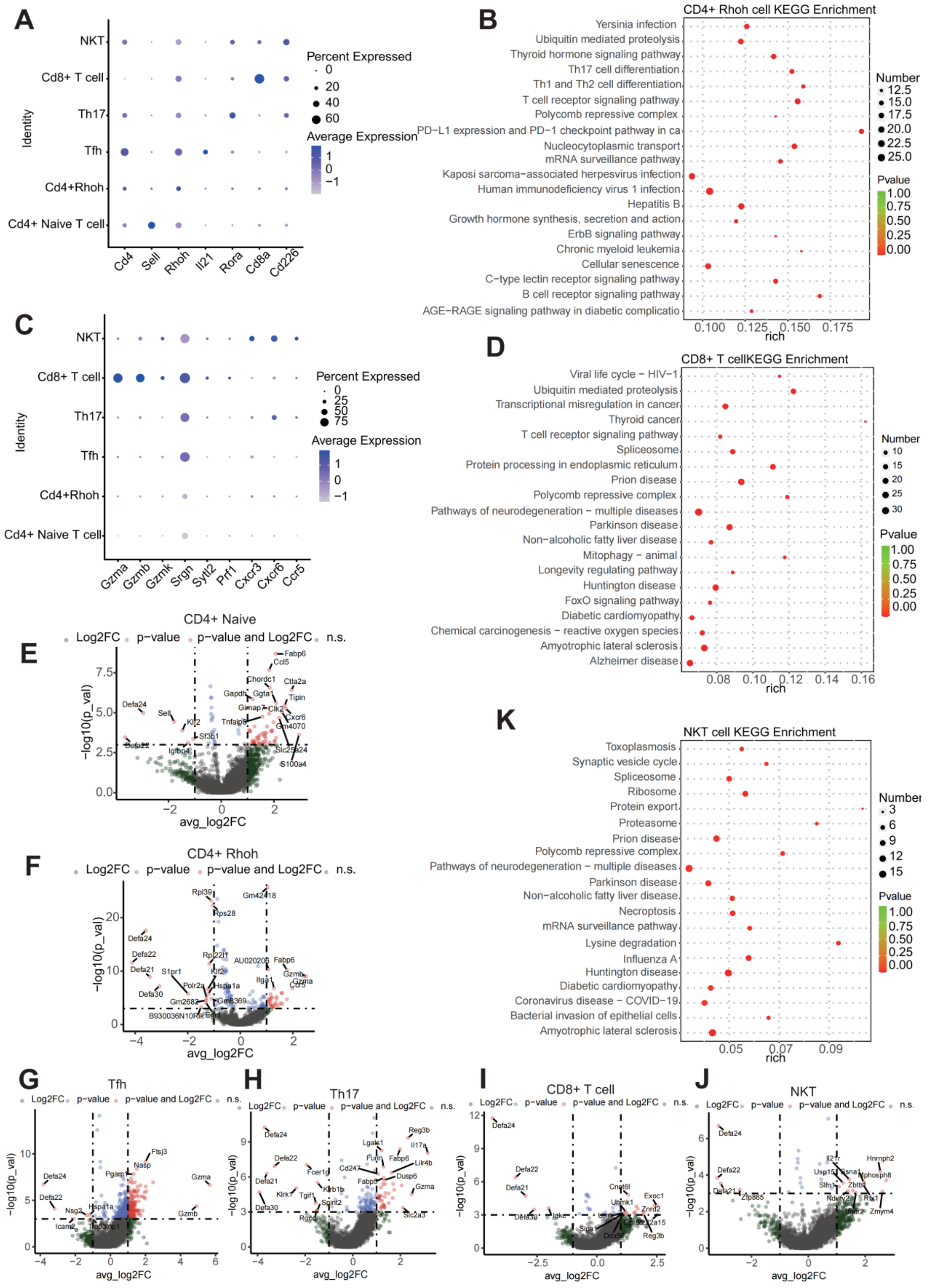

Figure S2 Characteristics of T cell subtypes, related to Figure 5. (A) Distribution and

expression of representative selected genes in clusters from Fig. 5A. (B) KEGG enrichment analysis of CD4$^+$ Rhoh cell based on DEGs among T cell subtypes. (C) Distribution and expression of cytotoxin and chemokine genes in clusters from Fig. 5A. (D) Bubble chart showing the enriched pathways in CD8$^+$ T cells in F gruop, by KEGG based on up-regulated genes. Volcano plot showing differentially expressed genes between Ctrl and fluoride-exposed CD4$^+$ naive T cell (E), CD4$^+$ Rhoh (F), Tfh (G), Th17 (H), CD8$^+$ T cell (I), and NKT cell (J) subtypes. The names of the most significant genes are indicated in the plots. (K) Bubble chart showing the enriched pathways in NKT cells in F gruop, by KEGG based on up-regulated genes.

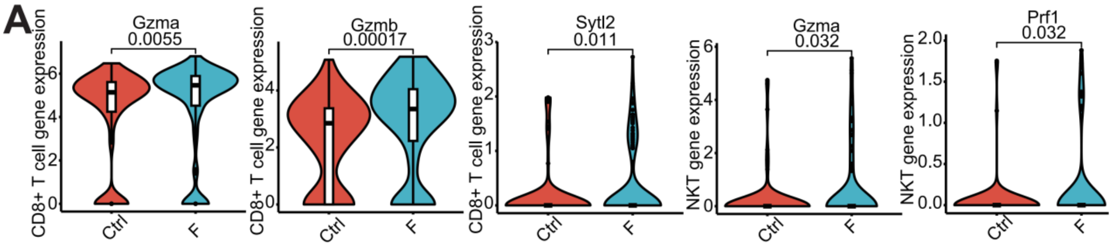

Figure S3 Expression characteristics of cytotoxic genes in in CD 8$^+$ T cell and NKT cell, related to Figure 5. (A) Violin plots show the significant up-regulated cytotoxic genes in CD 8$^+$ T cell and NKT cell in fluoride-exposed samples based on scRNA-seq. Significance was performed by using a paired Wilcoxon test.

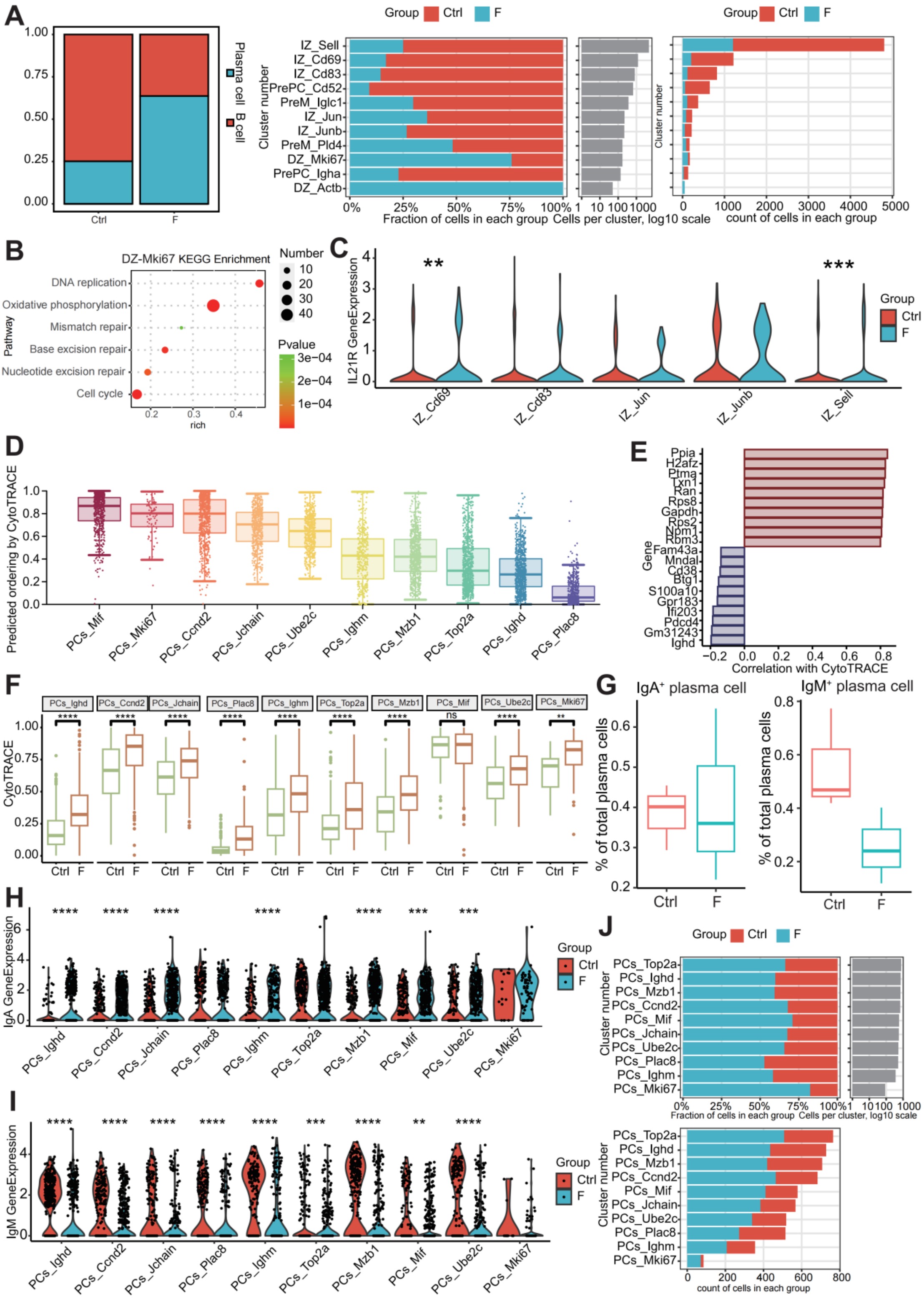

Figure S4 Characteristics of B cell and plasma cell (PC), related to Figure 6 and Figure 7. (A) Stacked bar chart of ratio of B cells and PCs in Ctrl and F groups (left). Difference in the percentage of cells (middle) and cell count (right) per B cell cluster (unexposed-exposed). (B) Bubble chart showing the enriched activated pathways in

DZ-Mki67 cells in the F gruop compared with the Ctrl group, by KEGG based on up-regulated genes. (C) Gene expression level of IL21R between F and Ctrl groups based on scRNA-seq. P-values were calculated using the paired Wilcoxon test. (D) CytoTRACE values showing the distribution of differentiation states from the most immature (highest values) to the most mature (lowest values). (E) Genes associated with the least (positive value) and most (negative value) differentiated cells based on CytoTRACE. (F) Differences of CytoTRACE scores of PCs subtypes between the Ctrl and F groups. P-values were calculated using the paired Wilcoxon test. (G) Histograms indicate the proportions of IgA+ and IgM+ plasma cell in total plasma cells. Gene expression levels of IgA (H) and IgM (I) in each PCs subtypes between F and Ctrl groups based on scRNA-seq. P-values were calculated using the paired Wilcoxon test. (J) Stacked bar chart of ratio of each PCs subtypes between Ctrl and F groups (Top). Cell number of each PCs between Ctrl and F groups (Down). *$p < 0.05$, **$p < 0.01$,***$p < 0.001$, ****$p < 0.0001$ were considered significant, and ns means no significant difference.

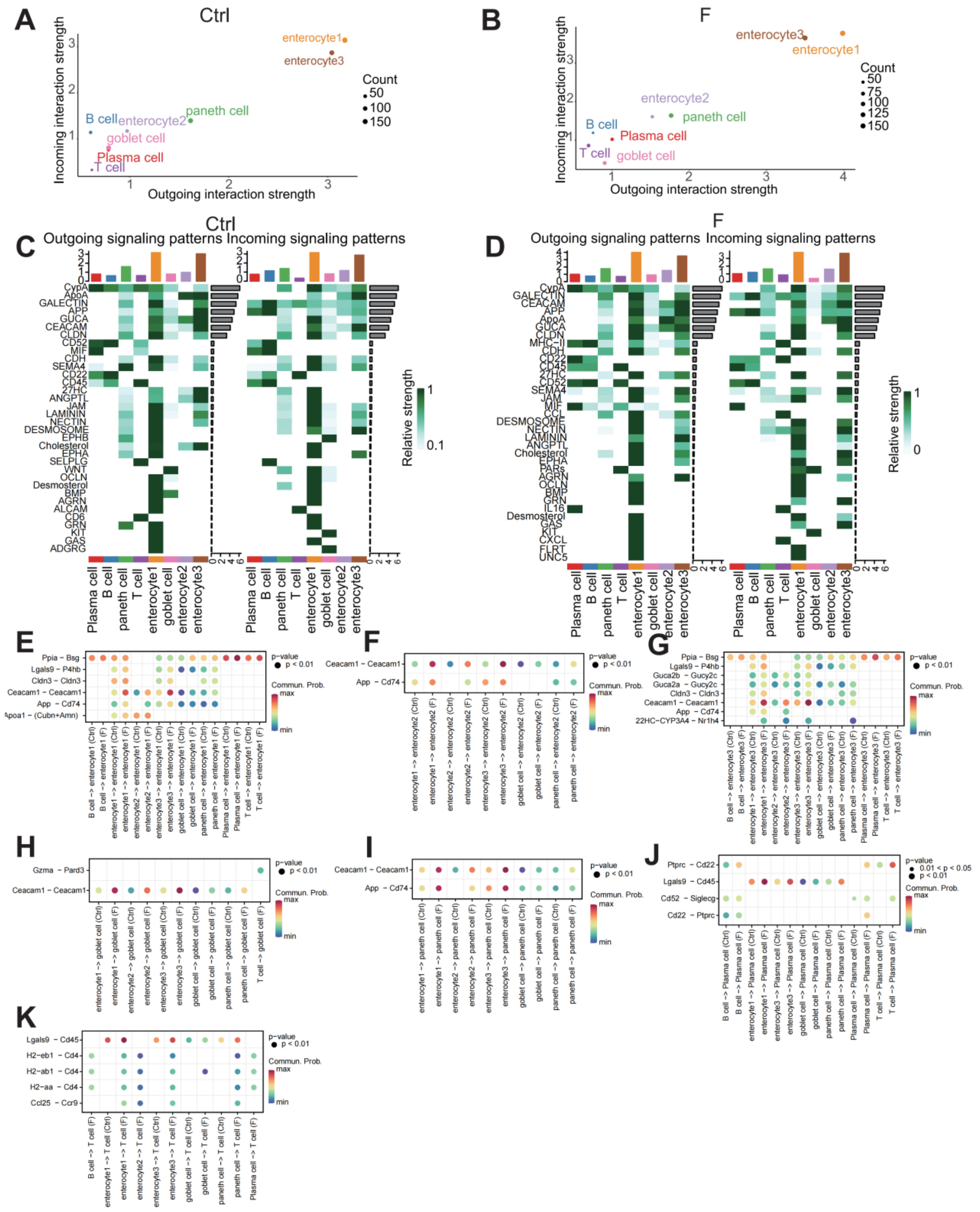

Figure S5. Cellchat analysis reveals a distinct intestine signalome in response to the fluoride exposure, related to Figure 8. (A) The incoming and outgoing strength of each structure and immune cell under normal intestine, respectively. (B) The incoming and outgoing strength of each structure and immune cell under fluoride-exposed intestine, respectively. (C) The outgoing and incoming signaling of each cell population in the normal intestine. (D) The outgoing and incoming signaling of each cell population in the fluoride exposed intestine. Bubble chart showing the

significantly enhanced interactions between enterocyte1 (E), enterocyte2 (F), enterocyte3 (G), goblet cell (H), paneth cell (I), plasma cell (J), and T cell (K) and their ligands in the F group compared with the Ctrl group. The sizes of the bubbles indicate the significance of the interactions, calculated by Cellchat.
